# Fair play? Participation equity in organised sport and physical activity among children and adolescents in high income countries: a systematic review and meta-analysis

**DOI:** 10.1186/s12966-022-01263-7

**Published:** 2022-03-18

**Authors:** Katherine B. Owen, Tracy Nau, Lindsey J. Reece, William Bellew, Catriona Rose, Adrian Bauman, Nicole K. Halim, Ben J. Smith

**Affiliations:** 1 SPRINTER, Prevention Research Collaboration, Level 6, Charles Perkins Centre, School of Public Health, Faculty of Medicine and Health, The University of Sydney, Sydney, NSW 2006 Australia; 2 Prevention Research Collaboration, Sydney School of Public Health, The University of Sydney, Sydney, NSW Australia; 3 The Australian Prevention Partnership Centre, Sydney, NSW Australia; 4 Centre for Healthcare Resilience and Implementation Science and the NHMRC Partnership Centre for Health System Sustainability, Australian Institute of Health Innovation, Macquarie University, Sydney, NSW Australia

**Keywords:** Socioeconomic position, Socioeconomic inequalities, Socioeconomic disparities, Physical activity, Sport, Children, Adolescents, Systematic review, meta-analysis

## Abstract

**Background:**

Physical activity and sport have numerous health benefits and participation is thought to be lower in disadvantaged children and adolescents. However, evidence for the disparity in physical activity is inconsistent, has not been reviewed recently, and for sport has never been synthesised. Our aim was to systematically review, and combine via meta-analyses, evidence of the socioeconomic disparities in physical activity and sport participation in children and adolescents in high income countries.

**Methods:**

We conducted searches of five electronic databases using physical activity, sport, and socioeconomic disparity related terms. Two independent reviewers assessed 21,342 articles for peer-reviewed original research, published in English that assessed socioeconomic disparities in physical activity and sport participation in children and adolescents. We combined evidence from eligible studies using a structural equation modelling approach to multilevel meta-analysis.

**Results:**

From the 104 eligible studies, we meta-analysed 163 effect sizes. Overall, children and adolescents living in higher socioeconomic status households were more likely to participate in sport (OR: 1.87, 95% CIs 1.38, 2.36) and participated for a longer duration (*d* = 0.24, 95% CIs 0.12, 0.35). The socioeconomic disparity in the duration of sport participation was greater in children (*d* = 0.28, 95% CIs 0.15, 0.41) compared with adolescents (*d* = 0.13, 95% CIs − 0.03, 0.30).

Overall, children and adolescents living in higher socioeconomic status households were more likely to meet physical activity guidelines (OR: 1.21, 95% CIs 1.09, 1.33) and participated for a longer duration (*d* = 0.08, 95% CIs 0.02, 0.14). The socioeconomic disparity in the duration of total physical activity between low and high socioeconomic status households was greater in children (*d* = 0.13, 95% CIs 0.04, 0.21) compared with adolescents (*d* = 0.05, 95% CIs − 0.05, 0.15).

There was no significant disparity in leisure time physical activity (*d* = 0.13, 95% CIs − 0.06, 0.32).

**Conclusions:**

There was evidence of socioeconomic disparities in sport participation and total physical activity participation among children and adolescents. Socioeconomic differences were greater in sport compared to total physical activity and greater in children compared with adolescents. These findings highlight the need importance of targeting sport programs according to socio-economic gradients, to reduce inequities in access and opportunity to organised sport.

**Supplementary Information:**

The online version contains supplementary material available at 10.1186/s12966-022-01263-7.

## Background

Physically active lifestyles during childhood and adolescence are associated with a wide range of physical, mental and social benefits; these include improved physical fitness, cardiometabolic health, bone health, cognitive outcomes (e.g., academic performance), mental health (e.g., reduced symptoms of depression); and social benefits (e.g., improved self-esteem) [1]. Current evidence suggests that many of these benefits carry forward into adulthood [1, 2]. Despite the known benefits of physical activity, over 80% of adolescents do not meet the current recommendations for daily physical activity [3].

There is some evidence that socioeconomic status (SES) is associated with physical activity, in that people of high SES are more physically active than those of lower SES. Stalsberg and Pedersen [4] systematically combined evidence from 62 studies, published up to July 2009, that assessed the association between SES and physical activity in adolescents. The authors concluded that there was an association, and that adolescents with higher SES were more active than those of lower SES. However, results of individual studies were inconsistent, with 42% of studies reporting no or an opposite relation. Sallis, Prochaska [5] reviewed 54 studies on correlates of children’s physical activity and reported that parental SES and children’s physical activity were not associated in most studies. Similarly, Ferreira, Van Der Horst [6] conducted a large review on environmental correlates of physical activity, including socioeconomic status, among children and reported inconsistent findings. One possible explanation for these inconsistent findings is that these reviews combined studies assessing physical activity across all domains (i.e., total physical activity, leisure time physical activity, and sport).

To better understand the socioeconomic disparity in physical activity, we need to explore the disparity across different domains of physical activity. There is some evidence to suggest that children and adolescents from lower SES families participate in higher levels of leisure time activities, such as active play and walking, compared with children and adolescents from higher SES families (e.g., [7, 8]). This could be due to different facilitators and barriers experienced across children and adolescents in different SES groups [9]. For example, children and adolescents from high SES families might experience parental encouragement or pressure to prioritise academic tasks, rather than leisure activities. There is also some evidence to suggest that children and adolescents from lower SES families are less likely to participate in organised sport, compared with children and adolescents from high SES families (e.g., [10]). Children and adolescents from low SES families may face additional barriers to structured sports, such as the associated financial costs (e.g., registration fees and uniforms), transportation issues, and limited or poor availability of quality facilities and activities in the local neighbourhood and at school [9]. Children and adolescents from high and low SES families experience different barriers across different domains of PA, which may contribute to differing socioeconomic disparities across different physical activity domains.

Equity across the life course is a fundamental guiding principle in the World Health Organization’s Global Action Plan on Physical Activity (GAPPA), requiring countries to prioritise addressing disparities and reducing inequalities in their implementation of the action plan to achieve the proposed 15% reduction in physical inactivity in adolescents (and adults) by 2030 [11]. To address these disparities, we need a comprehensive understanding of the disparities across physical activity domains. This review aims to provide an up-to-date synthesis of studies concerning socioeconomic differences in physical activity and organised sports participation among children and adolescents in high income countries. The rationale for this review, and the case for a value-adding contribution is as follows: (a) the need to examine and compare differentials in participation in organised sport as a distinct component of physical activity and (b) the equivocal or conflicting results of studies conducted since 2010.

## Methods

This systematic review and meta-analysis was registered at the Research Registry (ID: reviewregistry1147) and guided by the Preferred Reporting Items for Systematic Reviews and Meta-Analyses Statement [12].

### Eligibility criteria

To be included in this review, studies were required to:

- Examine children or adolescents (i.e., age range or mean age between 4 and 17 or enrolled in school).
- Not be limited to selected sub-groups (e.g., those with a medical condition, only overweight or obese, specific cultural or ethnic group).
- Quantitively assess sport participation, leisure time physical activity or total physical activity. Sport was defined as a structured activity through an organisation such as a club or school and leisure time physical activity was defined as any unstructured physical activity outside of school hours.
- In the case of leisure time and total physical activity, use population sampling at the first or second (depending on the country) subnational administrative level of the country. Due to the limited available data, this criterion was not applied to studies investigating organised sport participation.
- Use a quantitative measure of socioeconomic status (i.e., composite measure such as the Family Affluence Scale, household or parental income, parental education, neighbourhood socioeconomic status).
- Quantitatively assess socioeconomic differences in sport, leisure time physical activity or total physical activity.
- Use a cross-sectional, cohort or experimental (randomised controlled trials and quasi-experimental study design.
- Be conducted in one or more of the following countries: Australia, New Zealand, Canada, USA, UK, Switzerland, and member countries of the European Economic Area (EEA). Multi-country studies involving other countries, were eligible if they reported relevant data for the included countries. We recognise that there are also socioeconomic disparities in middle and low income countries [3], however there is evidence that physical inactivity is higher in high income countries and that the nature and scale of economic and social inequalities differ in high-, middle- and low-income countries [13]. For example, high-, middle- and low-income countries have different cultures of sport and non-organised physical activity and therefore, different barriers, correlates, and determinants, as well as a different distribution of SES. Further, there are limited data on sports participation available for low- and middle- income countries.
- Provide the full-text version in the English language.
- Be published between January 2010 and 15 July 2020.

### Information sources

Searches were conducted within Scopus, SportDiscus, PubMed, Medline and APA Psych Info in July 2020. Combinations of keywords were used to identify eligible studies.

### Search strategy

The search strategy combined terms relating to sport or physical activity, equity, and country limits. We developed the search strategy and validated it by testing whether it identified known relevant studies (e.g., [14, 15]. The full search strategy is presented in Supplementary Table 1.

### Selection processes

First, two researchers independently screened titles and abstracts for eligibility. Next, relevant full texts were retrieved and independently screened by two researchers. All discrepancies regarding inclusion criteria fulfillment were resolved by a third researcher.

### Data collection processes

Two researchers independently extracted data from eligible studies using a standardised extraction form. When the relevant data was not reported in the study, we contacted the corresponding author and requested the additional information.

### Data items

Extracted data included study characteristics (authors, year of publication, year of data collection, country in which the study was conducted), methods (study design, sample size, gender of participants), measurement (measure of sport, leisure time physical activity or total physical activity) and results (unadjusted and adjusted statistical results that examined the socioeconomic differences in sport, leisure time physical activity or total physical activity). In experimental and longitudinal studies with multiple timepoints, data was extracted from the first timepoint.

### Study risk of bias assessment

Risk of bias within studies was assessed using the Joanna Briggs Institute (JBI) critical appraisal instruments for studies reporting prevalence data, analytical cross-sectional studies, and cohort studies [16]. Two reviewers independently assessed each study, and any discrepancies were resolved by discussion between the two researchers or the consultation of a third reviewer.

### Effect measures

Commonly reported summary measures included means with standard deviations, standardised mean differences, regression coefficients, and odds ratios. All summary measures that assessed binary outcomes (i.e., participation in sport or meeting physical activity guidelines) were converted to odds ratios (comparing the lowest SES group with the highest). All summary measures that assessed continuous outcomes (i.e., duration of sport or physical activity participation) were converted to standardised mean differences (i.e., Cohen’s d; comparing the lowest SES group with the highest). Effect sizes were defined as small (OR = 1.68; d = 0.20), medium (OR = 3.47; d = 0.50), and large (OR = 6.71; d = 0.80) [17, 18]. There were 8 studies that did not provide the required information to convert the summary measure to an odds ratio or Cohen’s d. We contacted the 8 corresponding authors of these studies and 4 authors provided the additional information and so these studies were included in the meta-analyses. The other 4 studies could not be included in the meta-analyses.

### Synthesis methods

Typically, researchers have conducted meta-analyses using fixed-effects and random-effects models. However, these models are both limited by the assumption of independence, which means that only one effect size can be included per study [19]. To avoid violating the assumption of independence, researchers will a) average the effect sizes, b) “shift the unit of analysis” (i.e., retaining as many effect sizes as possible from each study while holding violations of the assumption of independence to a minimum), c) select one of the effect sizes or use a combination of the previously mentioned methods, or d) not report how the issue was handled [20]. These methods lose information and limit the research questions that can be answered and the ability to test moderators [21].

Two approaches to meta-analysis that are not limited by the assumption of independence are multilevel meta-analysis and structural equation modelling [22, 23]. These two approaches can be integrated to provide further methodological advantages [21]. The structural equation modelling approach to multilevel meta-analysis enables flexible constraints on parameters, constructs more accurate likelihood-based confidence intervals, and handles missing covariate data using full information maximum likelihood [21]. We took a structural equation modelling approach to multilevel meta-analysis. Unconditional mixed-effects models using maximum likelihood estimation were conducted to calculate the overall pooled effect sizes (pooled odds ratios and Cohen’s d’s). For each pooled effect size, 95% likelihood-based confidence intervals were calculated. All analyses were conducted using the metaSEM package [24] in R Version 4.1.1.

The *I*^2^ statistic was used to measure heterogeneity (i.e., variability in the effect sizes) [25]. An *I*^2^ statistic between 0 and 40% might not be important, 30 to 60% might represent moderate heterogeneity, 50 to 90% might represent substantial heterogeneity, and 75 to 100% considerable heterogeneity. These intervals overlap and so interpretations should depend on the magnitude and direction of the effect and the strength of the evidence for heterogeneity [25]. Heterogeneity can be examined and explained using moderator analyses.

We tested whether age moderated the socioeconomic differences in sport, leisure time physical activity and total physical activity. As sport dropout is highest during adolescence [26] and physical activity has the steepest decline during adolescence [27], we compared the socioeconomic differences in children (under 13) and adolescents (age 13 and above) [28, 29]. *R*^*2*^ was used to examine the proportion of variance explained by including age as a moderating variable.

We conducted a sensitivity analysis excluding studies that did not adjust for confounders to assess the role and extent of confounding [30].

### Reporting bias assessment

To examine reporting bias, we used funnel plots [31] and Egger’s regression asymmetry tests [32]. Funnel plots plotted the effect sizes on the x-axes and standard errors on the y-axes and resemble a symmetrical inverted funnel when there is no reporting bias. Egger’s regression asymmetry tests regress the normalized effect estimate (effect size divided by its standard error) against precision (reciprocal of the standard error of the effect size) and when the regression line runs through the origin, there is no reporting bias.

### Certainty assessment

The certainty of evidence was assessed using the Grading of Recommendations Assessment, Development and Evaluation approach [33]. Two researchers qualitatively assessed risk of bias, consistency, and precision and gave a summary rating – high, moderate, or low certainty of evidence.

## Results

### Study selection

**Fig. 1:**
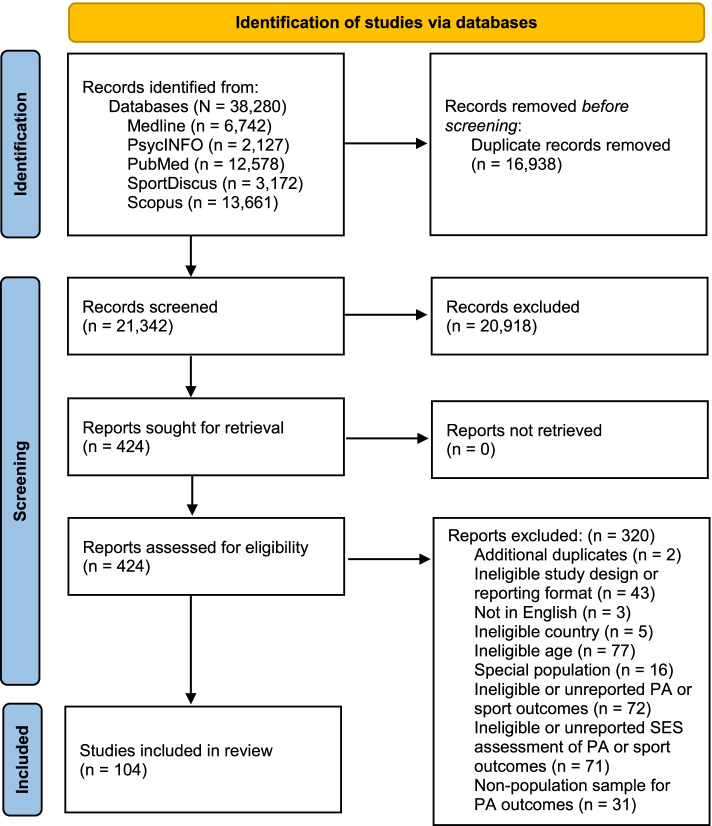
PRISMA flow diagram for study inclusion

Study selection results are presented in Fig. 1 (flow diagram). Through searches of electronic databases, we identified 21,342 non-duplicate records. After reviewing titles and abstracts, we obtained and reviewed full-text versions for 424 potentially relevant records. Of these 424 full text articles, 104 met the inclusion criteria. However, four of these did not provide enough information to be included in the meta-analyses.

We excluded 320 articles in the full-text review phase for the reasons identified in the PRISMA flow diagram (Fig. 1). These reasons included ineligible age (e.g. [34] was excluded because the mean age of the children in the analysis sample was 3.5 years); sample consisting of a ‘special population’ (e.g. [35] recruited student athletes); not reporting eligible physical activity or sport outcomes (e.g. the outcome reported in [36] was whether the person lived in a supportive neighbourhood for physical activity); not analysing the physical activity or sport outcome according to an eligible socioeconomic measure (e.g. [37] assessed physical activity according to weekly spending money). Any physical activity studies that did not use population sampling were excluded (or else only had their sport outcomes extracted) (e.g. [38] used convenience sampling to select high schools from Aveiro, a relatively small city and municipality in Portugal).

### Study characteristics

Study characteristics are detailed in Additional file 3. Of 104 included studies, 55% (k = 57) were published between 2010 and 2015 and 45% [39] were published between 2016 and 2020. Studies were conducted in Europe (k = 64 [Scandinavia k = 14; United Kingdom k = 12; other k = 38]), United States (k = 21), Australia or New Zealand (k = 12), and Canada (k = 7).

Across the 104 studies, there were 1,373,580 children and adolescents included. The number of study participants ranged from 200 [40] to 671,375 [41]. The mean age of study participants ranged from 4.7 years (SD = 0.9 [42];) to 17.0 years (SD = 0.9 [43];).

The majority of studies measured total weekly physical activity (k = 63), followed by sport (k = 40) and leisure time physical activity (k = 13; [12 studies assessed multiple outcomes]). Of the 63 studies that examined total physical activity, 18 studies used objective measures (accelerometers), and the remaining 45 used parent or self-report questionnaires. All studies assessing leisure time physical activity and sport used parent or self-report questionnaires.

Studies assessed socioeconomic status using income (k = 42), parental education (k = 26), a composite measure (e.g., Family Affluence Scale; k = 17), an area level indicator (e.g., Socioeconomic Indexes for Areas; k = 16) and eligibility for free lunch at school (k = 3).

### Risk of bias in studies

Complete risk of bias assessments are displayed in Additional file 4. The interrater agreement for risk of bias ratings was 76%, and all discrepancies were resolved by discussion between two researchers, or the consultation of a third reviewer where appropriate. Studies assessing sport participation met between 18 and 100% of risk of bias items (Mean = 66%). Studies assessing leisure time physical activity met between 62 and 91% of items (Mean = 73%) and studies assessing total physical activity met between 27 and 100% of items (Mean = 79%). Overall, the criteria that were least likely to be met were conducting the data analysis with sufficient coverage of the identified sample (k = 36 met this criteria) and measuring the outcome in a valid and reliable way (k = 57 met this criteria).

### Results of syntheses

#### Sport

Overall, children and adolescents living in higher socioeconomic status households were 1.87 times more likely to participate in sport (OR: 1.87, 95% CIs 1.38, 2.36, moderate certainty evidence; Table 1). Similarly, children and adolescents living in higher socioeconomic status households spent more time participating in sport (*d* = 0.24, 95% CIs 0.12, 0.35, low certainty evidence). For these pooled effect sizes, there was considerable heterogeneity between studies (*I*^*2*^ = 0.84 and 0.90, respectively) and negligible heterogeneity within studies (*I*^*2*^ = 0.15 and 0.10, respectively).

**Table 1:** Results of socioeconomic inequalities in physical activity and sport participation meta-analysis

Variable	# Studies	# ESs	ES	Lower 95% CI	Upper 95% CI	I^2^_2	I^2^_3	R^2^_2	R^2^_3	T^2^_2	T^2^_3
**Sport participation**											
Participation	**23**	**39**	**1.87**	**1.38**	**2.36**	**0.15**	**0.84**	0.02	0.00	0.20	1.00
Children	14	17	2.03	1.41	2.65						
Adolescents	7	19	1.84	1.14	2.55						
Duration (minutes)	**17**	**23**	**0.24**	**0.12**	**0.35**	**0.10**	**0.90**	0.00	0.05	0.00	0.04
Children	11	14	0.28	0.15	0.41						
Adolescents	5	7	0.13	−0.03	0.30						
**Total physical activity**											
Meeting guidelines	**31**	**37**	**1.21**	**1.09**	**1.33**	**0.34**	**0.56**	0.00	0.00	0.05	0.32
Children	16	19	1.07	0.76	1.38						
Adolescents	9	10	1.33	0.94	1.73						
Duration (minutes)	**38**	**56**	**0.08**	**0.02**	**0.14**	**0.67**	**0.26**	0.01	0.30	0.02	0.01
Children	22	38	0.13	0.04	0.21						
Adolescents	12	13	0.05	−0.05	0.15						
**Leisure time physical activity**											
Duration (minutes)	**7**	**8**	**0.13**	**−0.06**	**0.32**	**0.30**	**0.70**				

*Note*. The summary measure for sport participation and meeting physical activity guidelines are odds ratios and duration in Cohen’s d. A Cohen’s d of 0.2 is interpreted as small, 0.5 represents medium and 0.8 a large effect size. I^2^_2 = heterogeneity at Level 2 (i.e., between effect sizes from the same study); I^2^_3 = heterogeneity at Level 3 (i.e., between studies). R^2^_2 = variance explained at Level 2 (i.e., between effect sizes from the same study); R^2^_3 = variance explained at Level 3 (i.e., between studies)

#### Sport across age groups

Age explained a small portion of the heterogeneity found within studies that examined the socioeconomic differences in sport participation (*R*^*2*^ = 0.02). Children living in higher socioeconomic status households were 2.03 times more likely to participate in sport (OR: 2.03, 95% CIs 1.41, 2.65), and adolescents living in higher socioeconomic status households were 1.84 times more likely to participate in sport (OR: 1.84, 95% CIs 1.14, 2.55).

Duration of sport participation was also moderated by age (*R*^*2*^ = 0.05). There was a small to moderate difference in the duration of sport participation between children living in low and high socioeconomic status households (*d* = 0.28, 95% CIs 0.15, 0.41). Whereas there was a small non-significant difference in the duration of sport participation between adolescents living in low and high socioeconomic status households (*d* = 0.13, 95% CIs − 0.03, 0.30).

### Total physical activity

Children and adolescents living in higher socioeconomic status households were 1.21 times more likely to meet physical activity guidelines (OR: 1.21, 95% CIs 1.09, 1.33, high certainty evidence). For this pooled effect, there was moderate heterogeneity between studies (*I*^*2*^ = 0.34) and within studies (*I*^*2*^ = 0.56). Children and adolescents living in higher socioeconomic status households spent more time participating in physical activity (d = 0.08, 95% CIs 0.02, 0.14, moderate certainty evidence). For this pooled effect, there was substantial heterogeneity between studies (*I*^*2*^ = 0.67) and negligible heterogeneity within studies (*I*^*2*^ = 0.26).

### Total physical activity across age groups

Differences between children and adolescents living in low and high socioeconomic households meeting physical activity guidelines was not moderated by age (*R*^*2*^ = 0.00).

Age explained a small portion of the heterogeneity found within studies that examined the socioeconomic differences in duration of total physical activity (*R*^*2*^ = 0.02). There was a small difference in the duration of total physical activity between low and high socioeconomic status households in children (*d* = 0.13, 95% CIs 0.04, 0.21), but not adolescents (*d* = 0.05, 95% CIs − 0.05, 0.15).

### Leisure time physical activity

Children and adolescents living in higher socioeconomic status households spent more time participating in leisure time physical activity (*d* = 0.13, 95% CIs − 0.06, 0.32, low certainty evidence); however, the confidence intervals crossed zero. For this pooled effect size, there was considerable heterogeneity between studies (*I*^*2*^ = 0.70) and negligible heterogeneity within studies (*I*^*2*^ = 0.30).

### Sensitivity analyses

Supplementary Table 2 presents the pooled effects of studies examining socioeconomic differences in sport and physical activity, excluding studies that did not adjust for confounders. There were no appreciable differences when excluding these studies.

### Reporting biases

**Fig. 2:**
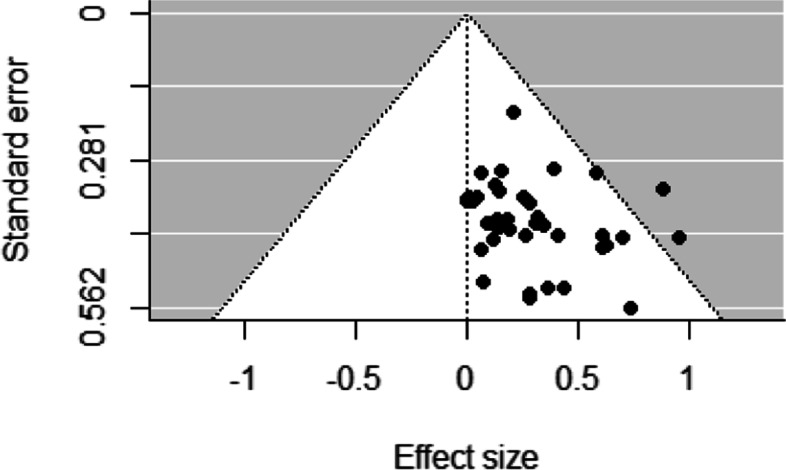
Funnel plot for sport participation

**Fig. 3:**
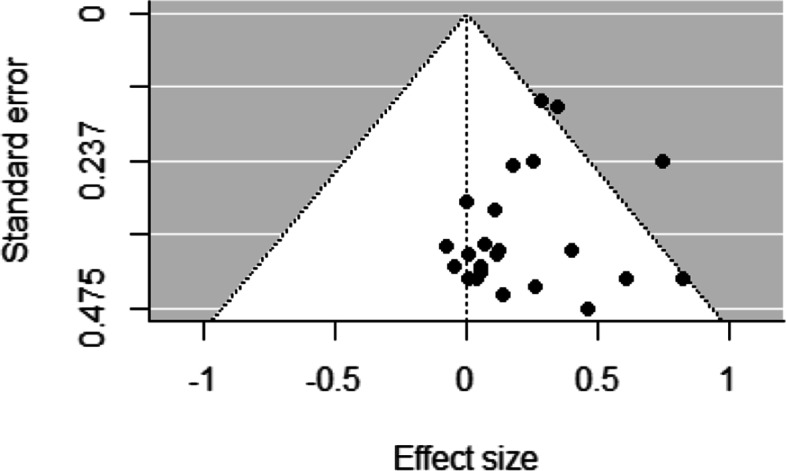
Funnel plot for sport duration

**Fig. 4:**
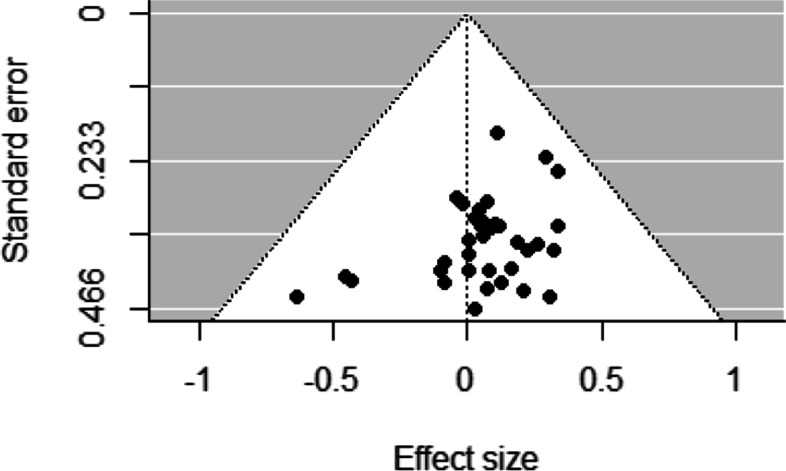
Funnel plot for meeting physical activity guidelines

**Fig. 5:**
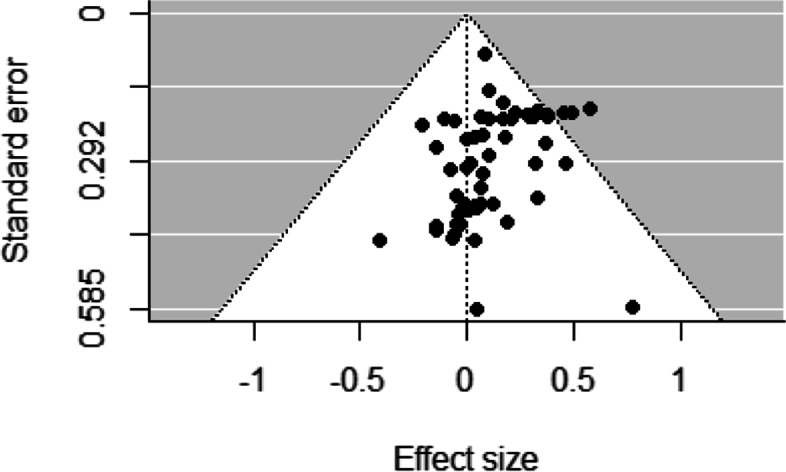
Funnel plot for total physical activity duration

**Fig. 6:**
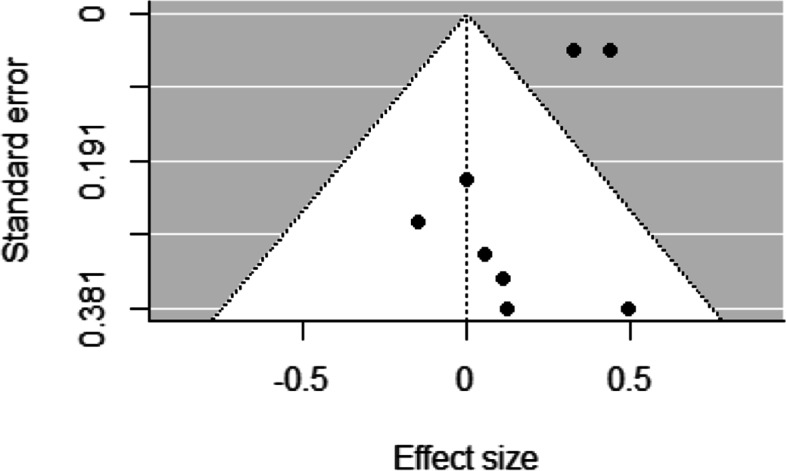
Funnel plot for leisure time physical activity duration

Funnel plots for studies examining socioeconomic differences in sport participation and duration of sport participation revealed low asymmetry, representing a low risk of bias across studies (Figs. 2, 3, 4, 5 and 6). This was confirmed by non-significant Egger’s test results (z = 1.34, *p* = 0.18 and z = − 0.80, *p* = 0.42, respectively). Similarly, funnel plots for studies examining socioeconomic differences in duration of leisure time physical activity participation revealed low asymmetry, representing a low risk of bias across studies, and this was confirmed by non-significant Egger’s test results (z = − 0.50, *p* = 0.62).

There was some evidence of risk of bias across studies that examined socioeconomic differences in meeting physical activity guidelines and duration of total physical activity participation. Funnel plots for studies examining socioeconomic differences in meeting physical activity guidelines and duration of total physical activity participation revealed moderate asymmetry, representing some risk of bias across studies. This was confirmed by significant Egger’s test results (z = − 2.63, *p* = 0.01 and z = − 2.67, *p* = 0.01, respectively).

### Certainty of evidence

The certainty of evidence for the socioeconomic differences in sport, leisure time physical activity and total physical activity are displayed in Table 2.

**Table 2:** Certainty of evidence for socioeconomic inequalities in sport and physical activity participation

Variable	# Studies	n	Findings	Certainty of evidence
**Sport participation**				
Participation	23	815,544	Odds ratio, 1.87 (1.38, 2.36)	Moderate certainty for small socioeconomic difference
Duration (minutes)	17	31,141	Cohens d, 0.24 (0.12, 0.35)	Low certainty for small socioeconomic difference
**Total physical activity**				
Meeting guidelines	31	1,073,470	Odds ratio, 1.21 (1.09, 1.33)	High certainty for small socioeconomic difference
Duration (minutes)	38	112,256	Cohens d, 0.08 (0.02, 0.14)	Moderate certainty for small socioeconomic difference
**Leisure time physical activity**				
Duration (minutes)	7	366,020	Cohens d, 0.13 (− 0.06, 0.32)	Low certainty for no socioeconomic difference

## Discussion

Since the late 1970s, equity in the context of health has become a central objective for the World Health Organization (WHO), largely attributed to the Alma-Ata Declaration of 1978, which emphasised the unacceptable nature of gross global health inequality and called for health for all by the year 2000 [44]. A little over a decade after the declaration, the WHO commissioned a definition of inequity that has come to be widely cited globally: “differences which are unnecessary and avoidable, but in addition, are considered unfair and unjust” [45, 46]. In 2021, WHO published an advocacy brief calling for stronger multisectoral action to address inequities in access and opportunities for physical activity [47]. Our systematic review and meta-analysis is the first to integrate evidence of the socioeconomic disparity in sport participation in children and adolescents. It is also the most recent and largest systematic review and meta-analysis of the socioeconomic disparity in physical activity. This included 104 studies and 126 effect sizes, with results showing small socioeconomic disparities (i.e., children and adolescents from high SES families are more active) in both sport (low- to moderate-certainty evidence) and total physical activity (moderate- to high-certainty evidence), but not leisure time physical activity (low-certainty evidence). Socioeconomic differences appear to be greater in sport compared to total physical activity or leisure time physical activity, and greater in children compared with adolescents.

While our overall pooled effects suggested inequities in sport and physical activity, there was no significant disparity in leisure time physical activity. It is important to note that only seven of the included studies assessed leisure time physical activity and these studies included a broad range of ages, introducing a high level of heterogeneity. Inconsistent findings of socioeconomic disparities were also found in a recent umbrella review of socioeconomic determinants of physical activity across the life course [39]. Some reviews identified socioeconomic disparities in physical activity (e.g., [48]), while others did not (e.g., [6]). One of the reasons could be that different types of physical activity and sport show unique and distinct socioeconomic disparities [15]. For example, the disparity tends to be greater in niche activities such as canoeing and rock climbing, compared to more mainstream activities such as cricket and netball, but may also depend on whether they occur in structured or unstructured settings. Children and adolescents from high and low SES families may also experience different barriers and facilitators across different domains of physical activity [9]. These inconsistencies could also be explained by unassessed confounders, such as culture, social organisation, geographic location, and factors beyond the scope of this review. For example, different activity preferences and participation patterns vary across different geographic regions. Hulteen [49] found that young people in the Americas (Canada, Jamaica, United States, Brazil) prefer team sports which may be associated with higher participation costs (e.g., Lacrosse), whereas those from the Western Pacific (Australia, China, Japan, Hong Kong) prefer physical activities including many that can be undertaken at little to no cost (e.g., running and walking). Further, each country has their own distinct school system with their own specific curriculum requirements and extracurricular sporting opportunities.

The socioeconomic disparities identified in this review were greatest in sports participation. This can be explained by a combination of individual (e.g., self-efficacy, negative outcome expectations), household (material or social deprivation) and neighbourhood (e.g., access and proximity to facilities) factors [14, 15]. Cost is a barrier that is greater for sport participation. Sport has several additional costs such as registration, uniform, travel, and equipment, which can present greater barriers for children and adolescents from disadvantaged backgrounds. This cost barrier to sport has been addressed through financial incentive programs across the world [50] which have shown some promising findings (e.g., [51]). However, there are socioeconomic disparities in awareness and engagement in these programs and further targeted work is needed [52].

It is important that the socioeconomic disparities in sport be reduced. The United Nations has identified sport as an important contributor to sustainable development [53, 54]. Sport has economic benefits, providing employment and local development. It can bring individuals and communities together, bridging cultural and ethnic divides. For young people, sport participation can be beneficial for holistic development, physical and emotional health and building valuable social connections [55]. Sport can also provide a healthy alternative to harmful behaviours such as drug use and crime [56]. In order to reduce the socioeconomic disparity in sport participation, a systems-based approach is needed that combines upstream policy actions to improve the social, cultural, economic and environmental factors for sport, with downstream actions that focus on the individual [11, 47].

Our review found that the socioeconomic disparity was greater in sport compared to physical activity, but this finding is based on evidence that was of low- to moderate certainty. Fewer studies have examined socioeconomic disparities in sport participation and these studies tended to have a higher risk of bias. Of the 40 studies that examined socioeconomic disparities in sport participation, only 5 used a valid or reliable measure of sport and 20 had a representative population sample. We recommend the development of a standardised and validated measure of sport participation that assesses both the frequency and duration of participation. Further high-quality studies with large representative population samples are required that examine the socioeconomic disparity in sport participation.

The socioeconomic disparities in our review were greater for dichotomous variables (i.e., sport participation vs. no participation and meeting physical activity guidelines vs. not meeting guidelines) compared to the continuous variables (i.e., duration of sport and physical activity participation). This suggests that once initial engagement in sport is established, socioeconomic status has less influence on the duration or frequency of participation. This is consistent with a previous study that found that there was a socioeconomic disparity in overall sport participation, but the disparity in regular participation was small [15]. Population targeted work is needed to establish initial participation in sport for children and adolescents from disadvantaged backgrounds. In relation to physical activity guidelines, the small group of children and adolescents who do meet guidelines (approximately one in five [3, 57];) are a select and distinct group. This group tends to have a higher level of advantage [58] and therefore, differentials could appear greater. Targeted work is needed to enable children and adolescents from disadvantaged backgrounds to meet physical activity guidelines.

The socioeconomic disparities in sport and physical activity were found to be greater in children compared with adolescents. This could be due to parental influence on physical activity and sport participation decreasing with age. As the child grows older, they gain autonomy and independence from their parents and are exposed to new environments and influences [39]. For example, as adolescents spend less time at home, and more time at school and with peers, the school environment and their peers become more influential in shaping health behaviours [39]. As such, parental SES (e.g., income or education) have reduced effect upon adolescents physical activity participation. There is evidence to suggest that alternate measures of socioeconomic status such as adolescent’s perception of social status relative to others in their peer group may be a better predictor of their health behaviours compared to the traditional measures [59]. Future studies should employ alternate measures of social status to further clarify the SES patterns for adolescents’ physical activity and sport participation. Disparities may also be lessened by the trends towards dropout in sport among adolescents [26] and the steep decline in physical activity across all socioeconomic groups in this age group [27]. Competing priorities (e.g., academic achievement), and increased responsibilities including schoolwork and employment begin to influence adolescents at this stage and will affect this age group differently across diverse social and cultural contexts internationally.

The differences in participation discussed here surely meet the WHO definition of inequity as “differences which are unnecessary and avoidable, but in addition, are considered unfair and unjust”; these inequities could be reduced by the right mix of government policies [60]. Progress requires a coordinated and strategic systems approach as outlined in the WHO Global Action Plan on Physical Activity 2018–2030 [11] and in the 2021 WHO advocacy brief Fair Play [47] which places particular emphasis on three areas of action (i) innovative and diverse financing mechanisms; (ii) coherent policy, laws, regulatory frameworks, and standards; and (iii) more integrated delivery of physical activity. These three domains indicate the need for progressing a research agenda that can determine whether the state and non-state actors engaged in the delivery of GAPPA have risen to these challenges.

### Limitations

A limitation of this study is that there was considerable heterogeneity in the pooled effect sizes. Some of this heterogeneity could be attributed to the variety of socioeconomic status measures (e.g., household income, parental education, area level socioeconomic status) [61]. Second, there was some publication bias in the meta-analysis of evidence pertaining to the socioeconomic disparity in physical activity. This is expected when conducting searches of published literature [62]. However, the meta-analysis also included null findings, suggesting that publication bias is likely not severe. Third, our searches may have missed relevant studies as they were limited to full text, conducted in high income and generally Western countries, in the English language between January 2010 and 15 July 2020. Further, the findings of our systematic review and meta-analysis are only relevant to high income Western countries. Further research is needed to investigate the socioeconomic disparities in sport and physical activity in children and adolescents living in low- and middle-income countries. Fourth, when we examined age as a moderator, we divided studies into two categories (children and adolescents). While we used a mean age of 13 as a basis for classification (i.e., younger than 13 years for children, and 13 or older for adolescents), it is likely that studies had participants in both categories. Although this method is limited, it does provide some understanding of how the socioeconomic disparity is different in children and adolescents.

## Conclusion

This systematic review and meta-analysis found evidence of small socioeconomic disparities in sport participation and total physical activity participation among children and adolescents in high income countries. Socioeconomic differences were greater in sport compared to total physical activity and greater in children compared with adolescents. These findings highlight the need to integrate an equity focus into programs and policies that are designed to increase sport participation, whilst also addressing inequities in access and opportunities for all children and adolescents to be physically active. Strategies to increase the equitable provision of positive and good quality physical activity and sport opportunities in childhood and adolescence, will help develop and strengthen the health and physical literacy skills needed to promote lifelong participation in sport and physical activity.

## Supplementary Information

**Additional file 1.**

**Additional file 2.**

**Additional file 3.**

**Additional file 4.**

## Data Availability

The data are available from the corresponding author on request.

## Abbreviations

EEA: European Economic Area
GAPPA: Global Action Plan on Physical Activity
JBI: Joanna Briggs Institution
OR: Odds Ratio
SES: Socioeconomic status
WHO: World Health Organisation
