# Supplementary material for: Fair play? Participation equity in organised sport and physical activity among children and adolescents in high income countries: a systematic review and meta-analysis
Source: Int J Behav Nutr Phys Act. 2022 Mar 18;19:27. doi: 10.1186/s12966-022-01263-7 (PMC8932332; doi:10.1186/s12966-022-01263-7)
Supplement: Supplementary file 1 — Additional file 1. [file 12966_2022_1263_MOESM1_ESM.docx]

# Final search strategy 14-15 July 2020

## **Search queries**

1. Sport related terms

“Sports**[MeSH Terms]”** OR “Sport” OR “Physical activity” OR “physically active” OR “Physical inactivity” OR “Physical fitness” OR “Exercise**[MeSH Terms]”** OR “Moderate-to-vigorous” OR “moderate to vigorous” OR “MVPA”

AND

1. Equity related terms

“Inequit*” OR “Equit*” OR “Disparit*” OR “Socioeconomic” OR “Socio-economic” OR “Socio economic” OR “Social class” OR “Disadvantage” OR “Depriv*” OR “SES” OR “income” OR “Inequality”

AND

1. Country limits [59 in total]

Australia*

“New Zealand”

“United Kingdom”

England

British

Scotland

Scottish

Wales

Welsh

Ireland

Irish

Canada

Canadian*

“United States”

“USA” [{USA} in Scopus]

America*

***Additional EU countries (plus EEA in bold + Switzerland)***

Austria*

Belgi*

Croatia*

Cyprus

Cypriot*

Czech

Denmark

Danish

Estonia*

Finland

Finnish

France

French

German*

Greece

Greek

Hungar*

**Iceland***

Italy

Italian*

Latvia*

**Liechtenstein**

Lithuania*

Luxembourg

Malta

Maltese

Netherlands

Dutch

**Norway**

Norwegian

Poland

Polish

Portug*

Romania*

Slovak*

Slovenia*

Spain

Spanish

Sweden

Swedish

**Switzerland**

Swiss

Europe*

## **Limits**

English language

AND

Published between January 2010 – June 2020
