# Supplementary material for: Fair play? Participation equity in organised sport and physical activity among children and adolescents in high income countries: a systematic review and meta-analysis
Source: Int J Behav Nutr Phys Act. 2022 Mar 18;19:27. doi: 10.1186/s12966-022-01263-7 (PMC8932332; doi:10.1186/s12966-022-01263-7)
Supplement: Supplementary file 2 — Additional file 2. [file 12966_2022_1263_MOESM2_ESM.docx]

Supplementary Table 1. Sensitivity results of socioeconomic inequalities in physical activity and sport participation meta-analysis removing studies that did not adjust for confounders

| Variable | # Studies | # ESs | ES | Lower 95% CI | Upper 95% CI | I^2^_2 | I^2^_3 |
| --- | --- | --- | --- | --- | --- | --- | --- |
| **Sport participation** |  |  |  |  |  |  |  |
| Participation | 17 | 32 | 1.79 | 1.25 | 2.32 | 0.16 | 0.82 |
| Duration (minutes) | 12 | 18 | 0.18 | 0.08 | 0.29 | 0.00 | 0.60 |
| **Total physical activity** |  |  |  |  |  |  |  |
| Meeting guidelines | 21 | 26 | 1.20 | 1.06 | 1.35 | 0.34 | 0.55 |
| Duration (minutes) | 28 | 34 | 0.06 | 0.01 | 0.12 | 0.08 | 0.41 |
| **Leisure time physical activity** |  |  |  |  |  |  |  |
| Duration (minutes) | 3 | 4 | 0.14 | -0.09 | 0.38 | 0.64 | 0.00 |

Note. The summary measure for sport participation and meeting physical activity guidelines are odds ratios and duration in Cohen’s d. I^2^_2 = heterogeneity at Level 2 (i.e., between effect sizes from the same study); I^2^_3 = heterogeneity at Level 3 (i.e., between studies).
