# Supplementary material for: Fair play? Participation equity in organised sport and physical activity among children and adolescents in high income countries: a systematic review and meta-analysis
Source: Int J Behav Nutr Phys Act. 2022 Mar 18;19:27. doi: 10.1186/s12966-022-01263-7 (PMC8932332; doi:10.1186/s12966-022-01263-7)
Supplement: Supplementary file 3 — Additional file 3. [file 12966_2022_1263_MOESM3_ESM.docx]

Table 1. Study characteristics

| Study | Year of publication | Year data was collected | Study design | Sample size | Mean age | Male/female | Country | Sport method of measurement | Physical activity method of measurement | Socioeconomic status measure |
| --- | --- | --- | --- | --- | --- | --- | --- | --- | --- | --- |
| Allen 2015 | 2015 | Wave 1 (2004) & wave 5 (2012) | Longitudinal/ cohort | 2301 (n=1722 43.5% not included in Table 1) | Wave 1: age 4-5 years; Wave 5: 12-13 years | 1176/1125 | Australia | LSAC Questionnaire - whether child had participated in team/individual sport in past week; if so, no. of days and average hours participating in that sport on those days. | | Household income |
| Amin 2018 | 2018 | 2015-2016 | Cross-sectional/ Prevalence | 968 | Grade 3-4 | 425/543 | United States | | Accelerometer (ActiGraph GT3X + and GT3X-BT models) | Eligibility for free/reduced lunch - Yes=Low SES; No=Middle SES |
| Andersen 2019 | 2019 | 2015 | Cross-sectional/ Prevalence | 10,531 | 17.0 (SD=0.9) | 4844/5687 | Norway | Questionnaire-˜How often do you exercise or take part in the following activities? Active participants were categorized as those who exercised at least 1-2 times a week. | | Family affluence was measured using four items from the Family Affluence Scale (FAS II) (Currie et al., 2008) |
| Armstrong 2018 | 2018 | 2007-2016 | Cross-sectional/ Prevalence | 3318 | 12-17yo | 55.2/44.8 | USA |  | NHANES questionnaire | Household income as ratio of federal poverty level |
| Bagordo 2017 | 2017 | 2014-2015 | Longitudinal/ cohort | 1164 | 7.34 ys (SD 0.87) | 50.9/49.1 | Italy | Questionnaire - designed for the MAPEC_LIFE study | | Parent combined education classified as <26 yrs, or 26+ yrs |
| Bann 2019 | 2019 | 2015 | Cross-sectional/ Prevalence | Australia Female 6459 Male 6449 Austria Female 3236 Male 3263 Belgium Female 4246 Male 4293 Bulgaria Female 2466 Male 2656 Canada Female 9440 Male 9240 Croatia Female 2848 Male 2591 Czech Republic Female 3242 Male 3221 Denmark Female 3230 Male 3120 Estonia Female 2727 Male 2679 Finland Female 2753 Male 2827 France Female 2811 Male 2600 Germany Female 2482 Male 2364 Greece Female 2558 Male 2574 Hungary Female 2684 Male 2612 Iceland Female 1648 Male 1502 Ireland Female 2713 Male 2762 Latvia Female 2345 Male 2322 Lithuania Female 3005 Male 3025 Luxembourg Female 2462 Male 2351 Montenegro Female 2415 Male 2374 Netherlands Female 2545 Male 2467 New Zealand Female 2062 Male 2038 Norway Female 2512 Male 2470 Poland Female 2148 Male 2170 Portugal Female 3424 Male 3370 Slovakia Female 2833 Male 3042 Slovenia Female 2779 Male 3229 Spain Female 3297 Male 3180 Sweden Female 2520 Male 2423 Switzerland Female 2609 Male 2794 United Kingdom Female 6396 Male 6513 United States Female 2668 Male 2626 | 15y6m to 16y2m | Australia 0.50/0.50 Austria 0.50/0.50 Belgium 0.50/0.50 Bulgaria 0.52/0.48 Canada 0.49/0.51 Croatia 0.48/0.52 Czech Republic 0.50/0.50 Denmark 0.49/0.51 Estonia 0.50/0.50 Finland 0.51/0.49 France 0.48/0.52 Germany 0.49/0.51 Greece 0.50/0.50 Hungary 0.49/0.51 Iceland 0.48/0.52 Ireland 0.50/0.50 Latvia 0.50/0.50 Lithuania 0.50/0.50 Luxembourg 0.49/0.51 Montenegro 0.50/0.50 Netherlands 0.49/0.51 New Zealand 0.50/0.50 Norway 0.50/0.50 Poland 0.50/0.50 Portugal 0.50/0.50 Slovakia 0.52/0.48 Slovenia 0.54/0.46 Spain 0.49/0.51 Sweden 0.49/0.51 Switzerland 0.52/0.48 United Kingdom 0.50/0.50 United States 0.50/0.50 | Australia Austria Belgium Bulgaria Canada Croatia Czech Republic Denmark Estonia Finland France Germany Greece Hungary Iceland Ireland Latvia Lithuania Luxembourg Montenegro Netherlands New Zealand Norway Poland Portugal Slovakia Slovenia Spain Sweden Switzerland United Kingdom United States |  | Non-validated PISA questions | Family wealth possessions (validated measure by Organisation for Economic Co-operation and Development - OECD). Separated by top, and bottom, quintile |
| Bengoechea 2010 | 2010 | 1998-99 | Cross-sectional/ Prevalence | 3159 | Range 12-15 years | 1626/1533 | Canada | Questionnaire: " In the last 12 months, how often have you played sports WITH a coach or instructor (swimming lessons, baseball, hockey, etc.)?" | | SES was a derived variable that included the parents’ education level, prestige of the parents’ occupation, and household income. |
| Boone-Heinonen 2010 | 2010 | 1994-1995 | Cross-sectional/ Prevalence | 17,294 | 11-22 years | 50.1/49.9 | USA |  | Interview administered, 7-item activity recall [based on questionnaires validated in other epidemiologic studies; no specific name of survey] | Advantageous socioeconomic environment as based on income, education and poverty |
| Borges 2015 | 2015 |  | Cross-sectional/ Prevalence | | 9-Nov |  | Portugal |  | Actigraph GT3X+ accelerometer | |
| Bringolf-Isler 2015 | 2015 | 2006-2009 | Cross-sectional/ Prevalence | 611 | 5.7 | 312/299 | Germany Switzerland | | Accelerometer | Highest parental education |
| Cairney 2015 | 2015 | 2004-2008 | Longitudinal/ cohort | Grade 4, Wave 1 = 2278; G5, W2 = 2228, W3 = 2273; G6, W4 = 2134, W5 = 2141, G7, W6 = 1896; G8, W7 = 1805. Total = 14,755 (more than one timepoint per participant) | 11.4 (0.3) | 7236 male/ 7059 female (more than one timepoint per participant) | Canada | The Participation Questionnaire (Hay 1992) | The Participation Questionnaire (Hay 1992) | Neighbourhood income |
| Cameron 2012 | 2012 | 2007 | Cross-sectional/ Prevalence | 4487 | 2-16 yrs | Not reported | Australia | | Multimedia Activity Recall for Children and Adolescents for 9-16yo Pedometers (New Lifestyles 1000) for 5-16yo | Parental income |
| Carroll-Scott 2013 | 2013 | 2009-2010 | Cross-sectional/ Prevalence | 753 | for overall sample but not the PA analysis specifically: 10.9 | for overall sample but not the PA analysis specifically: Boys: 549 (47.6%) Girls: 499 (52.4%) | USA |  | Patient-Centered Assessment and Counselling for Exercise (PACE) physical activity frequency item | Free/reduced lunch eligibility Food insecurity Neighbourhood SES |
| Clennin 2019 | 2019 | 2010, 2012 | Longitudinal/ cohort | 660 | 10.6 | 45.6%/54.4% | USA |  | Accelerometer (ActiGraph GT1M and GT3X models, Pensacola, FL, USA) | Neighbourhood socioeconomic deprivation |
| Collings 2014 | 2014 | 2005-06 | Cross-sectional/ Prevalence | 825 | 15 (SD=0.3) | 359/466 | England |  | sub-maximal step tests and Actiheart monitors (work for 4 days) | Area-level SES variable according to the classification of residential neighbourhoods (ACORN) index'. |
| DeCocker 2011 | 2010 |  | Cross-sectional/ Prevalence | 3352 | 14.8 (SD=1.2) | Male 47.6%/ Female 52.4% | Austria, Belgium, France, Germany, Greece, Hungary , Italy, Spain, Sweden | | | |
| DeCocker 2012 | 2012 | Not stated | Cross-sectional/ Prevalence | Total PA/ FAS- 2448  MVPA/ FAS- 2400 | 12.5- 17.49 yrs | males (47.1%); females (52.9%) | Austria, Belgium, France, Germany, Greece, Hungary , Italy, Spain, Sweden | | International Physical Activity Questionnaire- Adolescent (IPAQ-A) | Familial wealth index (measured using modified version of Family Affluence Scale (FAS)) |
| Deng 2018 | 2018 | 2015 | Cross-sectional/ Prevalence | 2123 | 9.20 (SD=1.75) | 50.4/49.6 | Norway |  | Accererometer (Actigraph wGT3X-BT, ActiGraph LLC, Pensacola, FL, USA) | Parental education [divided into primary school high school, bachelor's degree and master's/PhD degree] |
| D'Haese 2014 | 2014 | 2011-2013 | Cross-sectional/ Prevalence | 474 | 10.09 | 45.1%/54.9% | Belgium |  |  |  |
| Dmitruk 2014 | 2014 |  | Cross-sectional/ Prevalence | 1004 | Oct-18 | 53%/47% | Poland | Questionnaire developed by the author [no name given] | | Income per family member |
| Dollman 2010 | 2010 | 1985 / 2004 (?) - not clear whether current study reports on 1985 or 2004 survey data | Cross-sectional/ Prevalence | 1737 | not reported; children recruited from grades 5-10 | M: 45.3% F: 54.7% | Australia | The South Australian Physical Activity Survey | | Parental education (highest qualification achieved by the mother or father) |
| Duncan 2016 | 2016 | not reported | Cross-sectional/ Prevalence | 372 | 12.06 (SD=1.69) | 0%/100% | USA |  |  |  |
| Elgar 2016 | 2016 | 2010 | Cross-sectional/ Prevalence | 25,980 | 13.85 | 49.17/50.83 | Canada |  | Questionnaire: Physical activity was measured with the question: Over the past 7 days, on how many days were you physically active for a total of at least 60 min per day? with responses ranging from 0 to 7days. | Absolute deprivation between schools (based on HBSC Family Affluence Scale) Relative deprivation within schools (based on HBSC Family Affluence Scale) |
| Elinder 2014 | 2014 | 2009 | Experimental study | 1,220 | Mean age (years +/- SE):  Girls: Grade 2 cohort: 8.74 +/- 0.03 Grade 4 cohort: 10.81 +/- 0.03 Grade 7 cohort: 13.90 +/- 0.03  Boys: Grade 2 cohort: 8.77 +/- 0.02 Grade 4 cohort: 10.90 +/- 0.02 Grade 7 cohort: 13.91 +/- 0.03 | Male: 33.4% Female: 66.6% | Sweden | Questionnaire | | Parental education |
| Evans 2012 | 2012 | 2002-2003 | Cross-sectional/ Prevalence | 1184 | 10.6y | Male: 48% female: 52% | Italy, Lithuania, Portugal, Germany, Switzerland, France, Slovakia, Hungary | | Questionnaire answered by parent. 1- never engaged in sports or physical exercise. 5 - frequently at an intense level | Income |
| Fakhouri 2013 | 2013 | 2009-2010 | Cross-sectional/ Prevalence | 1218 | 6-Nov | 50.9/49.1 | USA |  | National Health and Nutrition Examination Survey (NHANES), answered by proxy respondent (usually a parent) | Income (family income to poverty level ratio)   <130% FIPR 130-349 >350% |
| Falconer 2014 | 2013 | 2010-2011 | Cross-sectional/ Prevalence | 2737 | not reported; 54.2% were in Reception year (ages 4/5 yrs) and 45.8% were in Year 6 (ages 10/11 yrs) | girls 51% boys 49% | England |  | Self-administered (unnamed) questionnaire completed by parents | English Index of Multiple Deprivation (IMD) 2007 |
| Ferrar 2012 | 2012 | 2007 | Cross-sectional/ Prevalence | 2071 | 9-16 [mean age not reported] | gender breakdown not reported | Australia | Use-of-time data from the Multimedia Activity Recall for Children and Adults (MARCA) | Use-of-time data from the Multimedia Activity Recall for Children and Adults (MARCA) | Parent-reported household income |
| Galan 2014 | 2013 | 2006 | Cross-sectional/ Prevalence | 15,902 | 11-18 yrs (average not stated) | 47.2% / 52.8% | Spain |  | Health Behaviour in School-aged Children (HBSC) survey | Composite Family Affluence scale (3 categories) |
| Goisis 2016 | 2015 | 2006 (5 yo) and 2012 (11 yo) | Longitudinal/ cohort | Age 5: 11965 Age 11: 9384 | Age 5  Age 11 | 51.6%/48.4% | United Kingdom | | Frequency of sport/exercise (less than once, once or twice, three times or more per week) | Quintiles of family income |
| Gracia-Marco 2010 | 2010 | 2002 | Cross-sectional/ Prevalence | 2165 | 15.2 (SD=1.5) | 51.9%/48.1% | Spain (5 cities) | Single item: “Do you undertake any physical sporting activity after school?” | | Parent education |
| Hardy 2012 | 2012 | 2007 | Cross-sectional/ Prevalence | 1568 (grade6 484;grade8 487;grade10 597 | school grades 6 8 and 10 11.6 years, 13.6years, and 15.6 years, respectively | 49% male 768 800 female | NSW Australia | | Adolescent Physical Activity Recall Questionnaire | Household income |
| Heradstveit 2020 | 2020 | 2012 | Cross-sectional/ Prevalence | 10257 | 17 yrs | 47.3%/52.7% | Norway |  |  |  |
| Herzig 2012 | 2012 | 2010 | Cross-sectional/ Prevalence | | 11.6 | 289/267 | Sweden and 7 other countries (European ENERGY partners) | questionnaire | | parental education |
| Hunt 2019 | 2019 | 2003-2006 | Cross-sectional/ Prevalence | 3,551 | All: 13.1 +/- 3.86  Low income: 13.3 +/- 4.0  Medium income: 12.9 +/- 3.8  High income: 13.1 +/- 3.7 | [All] Male: 50.1% Female: 49.9%  [Low income] Male: 47.3% Female: 52.7%  [Medium income] Male: 51.1% Female: 48.9%  [High income] Male: 50.4% Female: 49.6% | USA |  | PA monitor (Actigraph accelorometer) | Income-poverty ratio (function of household income) |
| Iguacel 2018 | 2017 | 2007-2008 2009-2010 | Cross-sectional/ Prevalence | 13891 subjectively measured PA / objective T0 5892 T1 2285 | Range 4-11 years | 7034 50.6% male 6857 female 49.4% / | Cyprus, Estonia, Germany, Hungary, Italy, Spain Sweden Belguim | | questionnaire accelerometer | Income (multiple used so went with income - this also wasn't used in later tables had to stick with proportions) |
| Imhof 2016 | 2016 | 2014 | Cross-sectional/ Prevalence | 322 | 7.3 +/-0.4 | Male (48.6%); Female (51.4%) | Switzerland (city of Basel) | Lipid Research Clinics (LRC) PA questionnaire | | Household income |
| Jekauc 2012 | 2012 | 2003-2006 | Cross-sectional/ Prevalence | 4529 | 11.3 (SD 4.1) | 50.4%/49.6% | Germany | | Questionnaire | Composite SES measure of parental education, professional status and household income |
| Jekauc 2013 | 2013 | 2003-2006 | Cross-sectional/ Prevalence | 4529 | 11.3 years (SD = 4.1 years) | Male: 50.5% Female: 49.5% | Germany | Motorik Modul (MoMo) Physical Activity Questionnire (MoMo-PAQ) | | SES categorisation based on combination of parental education, parental occupation*, and household income. |
| Jerina 2018 | 2018 | 2010 | Cross-sectional/ Prevalence | 669 | 9.9 SD 0.8 | 48.8% boys 321 boys 348 girls | Slovenia |  | Quality of life survey Sallis and owen 2002 | SES - monthly income per person in the previous year. |
| JimÃ©nez-PavÃ³n 2012 | 2012 | 2010 | Cross-sectional/ Prevalence | 7213 | 11.7 SD 0.7 | boys 3443 girls 3770 | Seven European countries (Belgium, Greece, Hungary,the Netherlands, Norway, Slovenia and Spain | | Questionnaire developed for the ENERGY study | Parent-reported educational level |
| Kim 2017 | 2017 | 2012-2013 | Longitudinal/ cohort | 2670 | 5-Oct | 51.4/48.6 | USA |  | Parental reported completion of PA question in the Geographic Research on Wellbeing (GROW) survey | Two levels of SES measures: Poor/non-poor based on neighbourhood economic context assessed based on the neighborhood poverty concentration (proportion of families with income that was below the federal poverty level for the census tract in which the respondent resided), and within that neighbourhood-level classification - family income (% of federal poverty level: <=100%, 101-200%, 2011-300%, 301-400%, >400%) and mother's education (less than high school; high school graduate/GED; some college or above). [NOTE, also derived measures of neighbourhood inequality using GINI index but not extracted here due to hierarchy preference for individual level measures] |
| Kimbro 2016 | 2015 | 1998-1999 | Cross-sectional/ Prevalence | 18,850. US-born = 16,030; Europe/Canada = 280; Mexico = 950; Latin America = 330; Southeast Asia = 560; Northeast Asia = 180; Caribbean = 250; Mideast/Africa = 270 | US-born: 74.7 months  Europe/Canada: 74.7 months Mexico: 73.5 months Latin America: 74.2 months Southeast Asia: 73.5 months Northeast Asia: 73.8 months Caribbean: 73.3 months Mideast/Africa: 74.6 months | Average: 51.2% male. US-born= 51.4% male, Europe/Canada= 53.5%; Mexico = 52.8%; Latin America = 49.0%; Southeast Asia = 50.3%; Northeast Asia = 52.2%; Caribbean = 51.2%; Mideast/Africa = 49.4% | USA | (parent interview) | Guidelines: three or more days per week of vigorous exercise which causes rapid breathing, perspiration, and a rapid heart beat for 20 continuous minutes or more | US FEDERAL POLICY THRESHOLD https://www.healthcare.gov/glossary/federal-poverty-level-FPL/ |
| Kipping 2015 | 2014 |  | Longitudinal/ cohort | 2346 | (median age: 16 years 7 months) | (Not stated) | England |  | ALSPAC postal questionnaire | Household income   [Household equivalized income: consists of quintiles of household’s disposable income assessed when the child was aged between 2 and 4 years. Based on declared income, the measure incorporates additional income obtained through housing and council tax benefits and is then adjusted for family size and composition"] |
| Kivimaki 2018 | 2018 | 2011/12 | Longitudinal/ cohort | 3002 | 10.9 (SD: 4.4) years | Male: 48% Female: 52% | Finland |  | (Unnamed study-specific questionnaire; question item not provided) | Childhood individual socioeconomic disadvantage was based the length of the parent's education (in years for the parent with the highest education), mean household income (continuous variable) and unemployment of the parent or parents during the follow-up (yes vs no)... |
| Kneeshaw-Price 2013 | 2013 | 2007-2009 | Longitudinal/ cohort | 682 | 9.1 (SD: 1.6) | Male: 50.2% Female: 49.9% | USA |  | MTI GT1M Actigraph accelerometer | Household income (<$50k, $50-100k, >$100k) |
| Kobel 2015 | 2015 | 2010 | Other: Cross-sectional analytic (baseline data from a cohort study) | 1714 | 7.1 +/- 0.6 years | Male: 50.3% Female: 49.7% | Germany | KiGGS survey questionnaire | KiGGS survey questionnaire | |
| Kowaleski-Jones 2017 | 2017 | 2003-2006 | Longitudinal/ cohort | 1584 | Mean age of sample not provided.   Age range across both relevant samples: 6-17 years | Total (across both age range groups): M: 50.1% F: 49.9%  [6-11 yo sample] M: 48.5% F: 51.5%  [12-17 yo sample] M: 51.3% F: 48.7% | USA |  | Accelorometer (Actigraph 7164;, LLC) | Income-to-poverty ratio   [Defined as "a ratio of a family’s total income to the appropriate poverty threshold given the family’s size and composition) Missing data on the income to poverty ratio were imputed using mean value with a random component added" |
| Krist 2017 | 2017 | 2010-2011 | Cross-sectional/ Prevalence | 1523 | 12.4 +/- 0.5 | Male: 49.5% Female: 50.5% | Germany | | Health Behaviour in School-aged Children (HBSC) questionnaire | FAS |
| Labree 2014 | 2014 | 2008/2009 | Other: Cross-sectional analytic (one wave only of a cohort study) | 1943 | Age range of participants: 8-9 years  Mean age ± SD (per migrant background category; overall mean not provided) [Dutch= 8.2 ± 0.45] [Turkish= 8.6 ± 0.67] [Moroccan= 8.5 ± 0.61] [non-western= 8.4 ± 0.63] [western= 8.3 ± 0.52] | Pooled m/f %: Male: 49.9% Female: 50.1% | The Netherlands | |  | Parental education |
| Lammle 2012 | 2012 | 2003-2006 | Cross-sectional/ Prevalence | 2574 | 11.51 +/- 3.36 | Male: 50.3% Female: 49.7% | Germany | | MoMo questionnaire | Household income |
| Lampinen 2017 | 2017 | 2007-2009 | Other: Cross sectional descriptive study | 486 | Girls= 7.6 +/- 0.4 Boys= 7.7 +/- 0.4 | Male: 51.0% Female: 49.0% | Finland | PANIC Physical Activity Questionnaire | PANIC Physical Activity Questionnaire | |
| Langlois 2017 | 2017 | 2006/2007 | Other: Cross sectional analytic | 2523 | (Mean age not stated) Age range of study participants: 14-18 years | M: 49.8% F: 50.2% | France | Boire Manger Bouger (BMB; Drinking, Eating, Moving) questionnaire | IPAQ (French short version, adapted for adolescents) | 1. Social and professional class of the family head categorised on the basis of three groups 2. Adolescent perception of family income (low or average; high 3. Residence area (urban or rural) |
| Lehto 2018 | 2018 | 2015-2016 | Cross-sectional/ Prevalence | 726 | 4.7 years (SD: 0.9) | M: 52% F: 48% | Finland |  | Accelerometer (Actigraph wGT3X-BT)  ["Children wore accelerometers for seven days, 24 h per day. Parents marked in a diary the hours children had spent at preschool as well as possible non-wearing hours of the accelerometers. | Parent education level |
| Love 2019 | 2019 | 2008-2009 | Other: Cross sectional analytic study (at age 7) - third phase of the cohort when accelerometer data were collected | 5172 | 6.8 +/- 0.4 years | Male: 49.8% Female: 50.1% | UK (England, Wales, Scotland, Northern Ireland) | | Accelerometer | Maternal education and equivalized household income (see top of page 3) |
| Macniven 2020 | 2020 | 2018 | Cross-sectional/ Prevalence | 671,375 | 4-18 years | Male: 53.9% Female: 46.1% | Australia | (Unnamed single-item sports question) | (Unnamed single-item PA question) | SEIFA |
| Manz 2016 | 2016 | 2003-2006 | Longitudinal/ cohort | 3471 | 8.5 years | M: 50.9% F: 49.1% | Germany | Frequency of organised sport participation was dichotomised into Y/N organised sport participation:  [In KiGGS0, sport participation was assessed via a self-administered questionnaire completed by a parent. The parent was asked how often her/his child participates in sports club activities with the response categories: almost every day; 3-5 times per week; 1-2 times per week; less than once per week; never. A dichotomous variable was also constructed. |  | Household income   [Household-equivalent income was calculated based on the households’ approximate monthly net income and the number of individuals living permanently in the household. |
| McCormack 2011 | 2011 | 2007 | Cross-sectional/ Prevalence | 927 | (Neither mean age nor age range of participants reported; authors state that participants included are from years 5-7) | Male: 45.7% Female: 54.3% | Australia |  | Pedometer  ["Accusplit (AH120 M8) pedometers were used to record children’s step counts... Children were asked to wear the pedometers at all times, except during water activities and while sleeping, for seven consecutive days including weekdays and weekend days"] | Parental education |
| McNeill 2017 | 2017 | 2006/2010 | Cross-sectional/ Prevalence | 1684 | (mean age not reported) 3-17 years | (Not stated) | Scotland |  | Scottish Health Survey (questions on Summary Activity Levels)   ["Questions on physical activity out of school, including walking, sport and exercise, active play, housework and gardening, and on time spent in front of a screen (television, computer or games console) out of school, were those used in the Scottish Health Survey"] | Socioeconomic Index of Multiple Deprivation (SIMD) score (area-level SES) |
| MiklÃNkovÃ 2016 | 2016 |  | Cross-sectional/ Prevalence | 200 | 5.71 ± 0.15 years | Male: 52% Female: 48% | Czech Republic | (Unnamed questionnaire) | | Parental (mother + father) education |
| Min 2018 | 2018 | 1998-1999 | Longitudinal/ cohort | 6440 | Not reported but PA data was collected when the children were in 8th grade [Baseline average kindergarten age was 6.3 yrs (SD=0.4)] | Not reported | USA |  | Child-completed PA question in the Early Childhood Longitudinal Study, Kindergarten Class (ECLS-K) questionnaire | Composite variable for household poverty dynamics, indicating whether the child lived in households with an income below, at or above federal poverty threshold during follow up. |
| Molina-Garcia 2017 | 2017 | 2013-15 | Cross-sectional/ Prevalence | 325 | 16.4 (SD=0.8) | 45.8/54.2 | Spain | Sport question developed for the IPEN Adolescent study. Question was: “In the past year, how many sports teams or after school physical activity classes (not physical education) have you participated in at school?” Response options were: 1, 2, 3 and 4 or more. | | Educational level of census blocks as obtained through the National Institute of Statistics for 2011 |
| Moore 2015 | 2015 | 2009-2010 | Cross-sectional/ Prevalence | 7823 | 11-16 years  (mean age of participants not reported in current study) | M: 50.2% F: 49.8% | Wales |  | "Children were asked on how many days in the previous week they participated in at least 60 minutes of physical activity. Children who selected more than 5 days were classed as physically active." | FAS   (NB. Free School Meal (FSM) entitlement also used as SES measure in current study, but not extracted here) |
| Morgan 2016 | 2016 | 2013-2014 | Cross-sectional/ Prevalence | 6499 for the PA analysis, 6216 for the MVPA analysis | 13.7 (SD=1.4) | 50.9/49.1 | Wales |  | Two self-reported PA questions: 1. students are asked “over the past 7 days, on how many days were you physically active for a total of at least 60 minutes per day?”  2. moderate to vigorous physical activity (MVPA) was assessed by asking students, “outside of school hours, how many hours in a week do you usually exercise in your free time so much that you get out of breath or sweat?” | Family Affluence Score [also analysed by school-level SES based on percentage of Free-school meal entitlement within each school - the school-level measure has not been extracted due to preference for individual level measures] |
| Morley 2012 | 2012 | 2009-2010 | Cross-sectional/ Prevalence | 12,188 | Dec-17 | 53/47 | Australia | | National Secondary Students' Diet and Activity (NaSSDA) survey which contains the following self-reported question: Over the past 7 days, on how many days were you physically active for a total of 60 minutes or more per day? with responses collapsed into 7 days, or not, to allow direct comparison with the PA recommendation. | SEIFA based on students' home postcodes, categorised into: low SEP (1st and 2nd quintiles) medium SEP (3rd and 4th) high SEP (5th) |
| Mulhall 2011 | 2011 | Not stated | Cross-sectional/ Prevalence | 1578 | 13 | 46/54 | USA | Self-reported weekly involvement in sports teams outside of school PE, broken down into categories of 1-2 d/wk, 3-4d/wk, 5-6d/wk, never and daily. [derived from CDC's Youth Risk Behavior Survey] | Self-reported exercise “how many of the past 7 days did you exercise or participate in a physical activity for at least 20 minutes that made you sweat and breathe hard”. The 6 specific activities given as examples are classified by the 2008 Guidelines as vigorous-intensity aerobic. | Student self-report of receiving either free or reduced-fee lunch services at school. Two categories: Free/reduced lunch; Neither |
| Nielsen 2012 | 2011 | (not stated) | Longitudinal/ cohort | 594 | 6.3 years (SD: 0.35) | M: 52% F: 48% | Denmark | (unnamed study-specific questionnaire)  "Questions on where the children did sports on a weekly basis were used to determine whether children were participating in organized sports or not. Children reporting institutional settings such as sports clubs, dance or riding schools were categorized as participating in club-organized sports."- see Materials and methods > measures of participation in organized sports (pp 7) |  | Socioeconomic position (indicators include combination of education level + parental occupation): |
| Nogueira 2013 | 2013 | Not reported | Cross-sectional/ Prevalence | 1885 | 3-Oct | 49.4/50.5 | Portugal | Questionnaire (unnamed) | | Years of parental education, categorised into three groups: <9 years (low-SES); 10-12 years (middle-SES); >12 years (high-SES). |
| Nogueira 2014 | 2014 | 2009-2010 | Cross-sectional/ Prevalence | 1885 | 3-Oct | 49.5/50.5 | Portugal | Unnamed questionnaire | | Years of parental education, based on father's education and categorised into three groups based upon the Portuguese educational system: children whose fathers have fewer than 9 years of education (i.e. the first three cycles of compulsory schooling) were considered low-SES; those whose fathers have 10 to 12 years of education (i.e. secondary schooling) were considered middle-SES; and those whose fathers have more than 12 years of education (i.e. post-secondary schooling) were considered high-SES. |
| Nyberg 2020 | 2020 | 2016-2017 | Cross-sectional/ Prevalence | 2236  (n is for relevant sample in Table 3, pp 9) | 14.4 years (SD: 2.6)   (^mean age of total sample, n=3477) | M: 41.3% F: 58.7%  (^M/F %s of relevant sample in Table 3, pp 9) | Sweden |  | Accelerometers Actigraph GT3X and GT3X+  ["Accelerometers Actigraph GT3X and GT3X+ were used to objectively measure physical activity among participants. The accelerometer was distributed during the school visit and participants were instructed to use them for 7 days except at night and when exposed to water. | Parental education |
| O'Brien 2020 | 2020 | 2014-2016 | Cross-sectional/ Prevalence | 15,040 | 5.27 years (SD: 0.31) | M: 51.6% F: 48.4% | Australia | | KHC questionnaire:  7. On about how many days during the school week does your child usually do physical activity outside of school hours?   8. On these days, about how many hours does your child usually do physical activity?  9. On about how many days during the weekend does your child usually do physical activity?  10. On a typical weekend day, about how many hours does your child usually do physical activity? | Area-level SES measure: Index of Relative Socio-Economic Disadvantage (IRSD), one of the Socio-Economic Indexes for Areas (SEIFA) |
| Pabayo 2011 | 2010 | 2001*-2006  *Only results from 2001 ("baseline", aged 10 years/5th grade) extracted here | Longitudinal/ cohort | 889 | 10.2 years | M: 50.1% F: 49.9% | US |  | Accelerometer- Computer Science and Applications (CSA) accelerometer  Physical activity across a typical week and weekend. | Income-to-needs ratio (function of family income); dichotomised into low income (<2.0) vs high income (>2.0) |
| Pearce 2019 | 2018 | 2005 (SES data); 2007 (PA data) | Longitudinal/ cohort | 6497  (column A in Table 1: all children w/ accelerometer data) | (exact mean age not stated, however can assume 7 years as PA data collected when participants were aged 7 years) | M: 51% F: 49% | UK (England, Wales, Scotland, Northern Ireland) | | Accelerometer (Actigraph GT1M; Pensacola, FL)   Daily moderate-to-vigorous physical activity (dMVPA) was defined as >2241 counts per min (cpm), and sedentary behaviour as <100 cpm. Time spent in dMVPA and sedentary activity was standardized to account for total valid wear time."] | Household income |
| Pitel 2013 | 2013 | 2006 | Cross-sectional/ Prevalence | 3217   NB. N above is for relevant PA analysis in tables 2 and 3 (total N of analytical study sample=3547) | 14.3 years (SD: 0.6) | M: 49.4% F: 50.6% | Slovakia |  | ["Physical inactivity was measured by the question: How many days per week are you usually physically active for more than 60 minutes? Respondents could choose the number of days, from 0 to 7. Those who were physically active fewer than 5 days per week were considered to be behaving unhealthily"]- see Methods > Instruments (pp 213) | Parental education   "highest educational level of the father and mother, categorized as 3 SEP levels: low (elementary school and apprenticeship), middle (completion of secondary school, including graduation examinations), and high (university education)." |
| Post 2018 | 2018 | 2016-2017 | Cross-sectional/ Prevalence | 941 | 13.0 (12.0-15.0) | 52.2/47.8 | USA | Survey (developed for this study) completed by parents which asked for the number of months per year and average number of hours per week that the child participates in their primary sport and in all organised sports in total. The child's primary sport was defined as the organized, competitive sport that is most important to the child. | | Total household income (THI) reported by parents categorically as either 0-50,000 USD per year, 50,001-100,000 USD per year, 100,001-200,000 USD per year, or >200,000 USD per year. |
| Poulain 2019 | 2019 | 2011-2017 | Longitudinal/ cohort | 3-10 yo: 182810-18 yo: 1488 | Mar-18 | not reported | Germany | Self-reported (if 10-18 yrs) or parent-reported (if 3-10 yrs) questionnaire by asking children (self-report) or their parents (parent report) how many times per week the child is physically active in sports clubs. For analysis, they compared children who were physically active at least once vs. less frequently than once per week. | | Index combining information on parental education (highest school degree and highest professional qualification), occupation (professional position) and income (family net income transformed to equivalent household income accounting for number of family members living in the same household). |
| Pouliou 2015 | 2014 | 2008-2009 | Longitudinal/ cohort | 6497 | 7 years   (all data are from children aged 7) | M: 50.9% F: 49.1% | UK |  | Accelerometer- [Actigraph GT1M uniaxial accelerometers (Actigraph, Pensacola, Florida)]  ["Children were instructed to start wearing their accelerometer the morning after receiving it and to do so for seven consecutive days during waking hours, except during bathing/aquatic activities... | Poverty (whether family income was <60% of national median)   ["Poverty was defined by whether family income was <60% of the national median, before housing costs but after benefits and using a modified Organisation for Economic Co-operation and Development (OECD) equivalence scale"]- see Methods > Explanatory variables > Home environment (reported measures) > The socioeconomic environment (pp 78) |
| PujadasBotey 2016 | 2015 | 2013 | Cross-sectional/ Prevalence | 623 [173 aged 2-4yo; 450 aged 5-13yo] | 8 (SD=4) | 52/48 | Canada |  | Parental reported questionnaire (Provincial Benchmark Survey, Alberta) which asked parents to identify the approx number of minutes per day that their child is physically active on a typical weekday. | Parent-reported annual income (<$40,000, $40,000-$99,999, > $100,000) |
| Puolakka 2018 | 2018 | 1980 (baseline data)  (follow-up data collected in 1983, 1986, 2007 and 2011- NOT extracted here) | Longitudinal/ cohort | 2213 | (mean age of relevant sample NOT reported)  Mean age of whole sample (n=3596, baseline) 10.4 years (SD: 5.0) | M: 49.1% F: 50.9% | Finland |  | Mothers were asked questions concerning their child’s outdoor play time in summer and in winter, the amount of PA in play as compared with other children, the vigorousness of PA, the child's enjoyment of indoor/outdoor play, the child's general level of activity as compared with other children, the encouragement given to participate in sports, and the patterns of PA. Each item was coded from 1 to 3, except for encouragement to engage in sport. By summing the variables, a PA index (PAI) of preschool children was formed with scores ranging from 8 to 23. The PA of 9- to 18-yr-old subjects in 1980 and 1986 was measured using a short self-report questionnaire administered individually in connection with a medical examination. The questions concerned the frequency and intensity of leisure time PA, participation in sports club training, participation in sport competitions, and habitual way of spending leisure time. The items were coded from 1 to 3 and summed to form a PAI with scores ranging from 5 to 15 (28).] | Annual household income (for children under 18 years) + participants' own income (for 18 year olds) |
| QunitoRomani 2020 | 2019 | 2008, 2010  [2008 reports 11-13 yo cohort data; 2010 reports 13-15 yo cohort data] | Longitudinal/ cohort | 1251   (NB. Above N is for OLS/2010 outcome data) | 6th form (2008) cohort: 11-13 years  8th form (2010) cohort: 13-15 years  (mean age not stated) | F: 49.1% M: 50.9 | Denmark | Participation in organised sports was captured by the question: Are you a member of a sports club? |  | Parental income |
| Quon 2015 | 2015 | 1999 | Cross-sectional/ Prevalence | 2199 (13 yo n=1049 16 yo n=1150) | 14.51 (SD=1.52) | 48.4 (1,065)/51.6 (1,134) | Canada |  | Seven-day recall adapted from the Weekly Activity Checklist (see Paradis 2003 citing Sallis 1993). For each day of the preceding week, subjects checked which of 18 physical activities they had participated in for at least 15 consecutive minutes on that day (which included physical education classes and activities during free play). A frequency score was computed by summing the total number of activities checked for each day of the week. | Household income measured by total household income (before tax) in the previous year as reported by parents, categorised according to different categorical bands and transformed into a continuous variable using the median value of each income category |
| Rao 2017 | 2017 | 2011, 2012, and 2014 | Cross-sectional/ Prevalence | Not reported | Range 5-17 | Not reported | Canada |  | Achieving age-specific PA guidelines of 60mins of moderate- to vigorous-intensity aerobic PA every day | Income adequacy: low, moderate, high |
| Rauner 2015 | 2015 | 2003-2006 | Longitudinal/ cohort | 934 | 11-17y. Young group (11-13y) and old group (14 -17y) at baseline only | 438 (46.9%)/496 (53.1%) | Germany | MoMo PhysicalActivity Questionnaire (MoMo-PAQ) | Overall physical activity (OPA) was assessed using a two-item questionnaire using MoMo-PAQ | Composite measure of parental educational and professional status and total family income |
| Reece 2020 | 2020 | 2015-2017 | Cross-sectional/ Prevalence | 7976 | 0-14y |  | Australia | AusPlay questionnaire, sport section | | The Australian Bureau of Statistics’ (ABS) Index of Relative Socio-economic Disadvantage (IRSD) |
| Rokicki 2019 | 2019 | 2007/2008 | Longitudinal/ cohort | 3923  ^N of analytical sample in wave 1 | (not stated, but can assume 9 years) | (not stated) | Ireland | Time use diary   ["Both waves of the GUI child cohort included a Time Use Diary (TUD), which recorded details on the activities of participants over a 24-hour period, dividing the day and night into 15-minute intervals"] | (sport/exercise were consolidated into one outcome "sports" in current paper; see above) | Household equivalised income |
| Ruiz 2011 | 2011 | 2006-2008 | Cross-sectional/ Prevalence | 2200 | Median (IQR)= 14.9 (13.9-15.8) years  Age range: 12.5 - 17.49 years | M: 46.2% F: 53.8% | Greece, Germany, Belgium, France, Hungary, Italy, Sweden, Austria, Spain | | Accelerometer (ActiGraph MTI GT1M; ActiGraph LLC, Pensacola, Florida) | Maternal education |
| Salvy 2017 | 2016 | 2012 - 2014  Wave 5 (2012) Wave 6 (2013-2014) | Longitudinal/ cohort | 2144 | 16.2 (0.74) years | M: 45.1% F: 54.9% | USA |  | [Participants reported the number of days in the previous week (0-7 days) that they were physically active for >60 min per day. | Neighbourhood SES (NSES)  [NSES- We geocoded adolescents€™ residential addresses to identify the census tract in which they lived. We then assigned each student a census tract-level median household income value using American Community Survey 5-year |
| Santinello 2012 | 2012 | 1997-1998 | Cross-sectional/ Prevalence | 3168 | 10.93/12.99 | 1669 (52.7%)/1499 (47.3%) | Italy | Questionnaire. "Outside school hours: how often do you usually exercise in your free time so much that you get out of breath or sweat?" and " Outside school hours: how many hours a week do you usually exercise in your free time so much that you get out of breath or sweat?". For the first question answers were given on a 7-point scale ranging from ''never" (1) to "every day" (7) and for the second question on a 6-point scale ranging from "none" (1) to "7 hours or more" | | Father's occupation, self-reported family affluence scale (no. of bedrooms/travel/car etc), subjective perception of family economic well-being |
| Shi 2014 | 2014 | 2008-2009 | Cross-sectional/ Prevalence | 2901 | 7-14y | 1559 boys (54%)/1342 girls (46%) | USA |  | Questionnaire | Annual household income. Merged into five groups:<$35k ,<$60k, <$80k,<$100k, and >$100k |
| Sigmundova 2019 | 2019 | 2006/2010/2014 | Cross-sectional/ Prevalence | 501,647  Ns per year: 2006= 167,176 2010= 172,707 2014= 161,764 | 13.59 ± 1.64 years | [2006] M: 48.4% F: 51.6%  [2010] M: 48.5% F: 51.5%  [2014] M: 48.4% F: 51.6%  [Overall] M: 48.4% F: 51.6% | Austria, Belgium/Flemish, Canada, Croatia, the Czech Republic, Denmark, Estonia, Finland, France, Germany, Greece, Greenland, Hungary, Iceland, Ireland, Israel, Italy, Latvia, Luxembourg, the Netherlands, Norway, Poland, Romania, Russia, Slovakia, Slovenia, Spain, Sweden, Switzerland, Ukraine, Macedonia, England, Scotland and Wales | | VPA was assessed by the two following items. Outside school hours: How often do you usually exercise in your free time so much that you get out of breath or sweat?  Outside school hours: How many hours a week do you usually exercise in your free time so much that you get out of breath or sweat? | Family Affluence Scale. |
| Smith 2015 | 2015 | 2012 | Longitudinal/ cohort | 3105 | 11-12 years  (mean age not stated) | M: 56.6% F: 43.4% | England |  | Youth-Physical Activity Questionnaire (Y-PAQ)   [Physical and sedentary activity was estimated by the self-reported Youth Physical Activity Questionnaire (Y-PAQ) [25]. This questionnaire assesses the accumulated time spent physically active or sedentary respectively over the previous seven days outside of school. | Household socioeconomic circumstances were quantified by the Family Affluence Scale. |
| Song 2013 | 2013 | 1996-2006 | Cross-sectional/ Prevalence | 6547 | Dec-17 | 51.1 (3,307)/48.9 (3,240) | USA |  | NHANES questionnaire  To assess aerobic activity, participants were asked about vigorous- and moderate-intensity activities engaged in at school or in leisure time over the previous 30 days. Participants who reported participating in at least 60 minutes of aerobic activity per day, 7days per week, met the recommendation for aerobic activity according to the 2008 Guidelines. | Family poverty-to-income ratio which is a variable representing the ratio of household income to the DHHS’s poverty threshold based on family size and adjusted for annual updates of inflation. The ratio was categorized into four groups (<100%, 100%-199%, 200-299%, and >=300%), where <100% is below the poverty threshold. |
| Steenholt 2018 | 2018 | 2014 | Cross-sectional/ Prevalence | 27,865 | (mean age of relevant sample not stated)  Mean age (SD) of total sample (N=70,674): 18 (1.6) years | (M/F% of relevant sample not stated)  M/F% of total sample (N=70,674): M: 39%; F: 61% | Denmark | Where do you perform sports and other forms of physical activity? Participation in organised PA was defined as having answered yes at school or in a sports association or club (e.g. football, dance, martial arts).] | Health Behaviour in School-aged Children (HBSC) PA questionnaire:   Outside of school: how many hours a week do you practice sport or other physical activity to the extent that you become breathless or sweat? | Parental education (national register data) |
| Tandon 2012 | 2012 | (not stated; only period of participant recruitment was identified: 2007-2009) | Longitudinal/ cohort | 713 | [Stratified by SES, according to household income, Table 1]: mean ± SD  Low SES= 9.2 ± 1.4 years Mid SES= 9.1 ± 1.6 years High SES= 9.1 ± 1.6 years | [Stratified by SES, according to household income, Table 1]: M%/F%  Low SES= 48%/52% Mid SES= 47%/53% High SES= 53%/47% | USA |  | Accelerometer assessed weekly physical activity | Household income   (ranging from<$10,000 to>$100,000) on the survey were combined into 3 categories each for analyses according to the following a priori criteria: Education- low (completed high school), medium (completed college), high (completed graduate degree); income - low ($39,000), medium ($40,000-$89,000), high ($90,000). |
| Taylor 2020 | 2019 | 2011 | Experimental study | 574 | 7.9 (1.1) years  (mean age of relevant sample not stated; above mean age is of total sample, n=690) | M: 48.5% F: 51.5%  (M/F proportions not provided for relevant sample; above proportions are of total sample, n=690) | New Zealand | | Accelerometer (ActiGraph GT3X, Pensacola, Florida, USA)  [Twenty-four-hour movement patterns were assessed by accelerometry. | Area-level deprivation index (The New Zealand Deprivation Index) |
| Uzochukwu 2017 | 2017 | 2011-2012 | Cross-sectional/ Prevalence | 25,094 | 13.6 years | M: 52% F: 48% | USA |  | [The physical activity variable measures the number of days in the past week the child exercised, played sports, or participated in physical activity for at least 20 min that made the child sweat and breathe hard; values range from 0 days to 7 days.]- see Measures (pp 424) | Household income |
| Vandermeerschen 2015 | 2015 | 2009 | Cross-sectional/ Prevalence | 2016 | 6-18 years | 50.6/49.4 | Belgium | Questionnaire asking for a binary response to: club-organised sport participation (yes/no) | | Net total income of the family |
| Vandermeerschen 2016 | 2016 | 1989/1999/2009 | Cross-sectional/ Prevalence | 2088 | 13-18 years  (mean age not stated) | M/F% per survey cycle:  [1989]: M: 50.8%; F: 49.1%  [1999] M: 48.8%; F: 51.2%  [2009] M: 50.6%; F: 49.4% | Belgium | Sport participation: students are considered as club participants if they declared to have practised at least one sport in a club-organised context during the last 12 months, regardless of the frequency, intensity or level of this sports practice. | | Parental education + socio-professional status |
| Vella 2014 | 2014 | 2008 & 2010 | Cross-sectional/ Prevalence | 4042 | 8.25 | 2069 (51%)/1973 (49%) | Australia | LSAC parent-report questionnaire. | | Standardised Household income |
| Vermeiren 2018 | 2018 | 2015 | Cross-sectional/ Prevalence | 530 | Not provided for the relevant analysis in Table 3 (a subset n=530 of the sample described in Table 1 n=784). For the larger sample (with information on deprivation but not necessarily valid PA data) - mean age was 8.0 (SD=2.3). | Not provided for the relevant analysis in Table 3 (a subset n=530 of the sample described in Table 1 n=784). For the larger sample (with information on deprivation but not necessarily valid PA data) - 48.2% male / 51.8% female | The Netherlands | | Accelerometer (Actigraph GT3X+, 30 Hz, processing by ActiLife 6.10.4) | Parental material deprivation measured by three binary items (I have enough money to . . . (i) heat my home (ii) pay for sport and club memberships (iii) visit friends/family), and one five-point item (meet an unexpected expense of 1000 euros) ranging from always to never). |
| Veselska 2011 | 2011 | 2006 | Cross-sectional/ Prevalence | 3694 | 14.3 | 49% (1765)/51% (1929) | Slovakia |  | Physical activity was assessed using a single question used and validated in Health Behaviour in School-aged Children (HBSC) surveys | Education: high (university), middle (secondary school), low (apprenticeship or primary school only) |
| Virtanen 2019 | 2018 | 2013 | Cross-sectional/ Prevalence | 76113 | 17 | 49% (36966)/51% (39147) | Finland |  | Questionnaire | Highest educational level of parents: basic education or less, upper secondary school/vocational education institution and university or other higher education institution |
| White 2012 | 2012 | 1998-1999 | Cross-sectional/ Prevalence | 18320 | 6-15 year |  | Canada | Multiple questions - parent response for 6-9 years, child response for 10-15 years | | Income (<15000, 15000-19999, 20000-29999, 30000-39999, >40000) |
| Wijtzes 2014 | 2014 | 2008-2012 | Longitudinal/ cohort | 4685 | 73.0 ± 5.9 months  (mean age of relevant sample not stated; above is mean age of total sample, N=4726) | M: 50.5% F: 49.5%  (M/F% of relevant sample not stated; above are proportions of total sample, N=4726) | the Netherlands | Single item: Does your child take part in sports (for example. football, judo, gymnastics, jazz ballet, tennis, etc.? Y/N"]- | | Household income  [... net household income (< 2000/month; 2000-3200/ month; >3200/month). |
