# Supplementary material for: Fair play? Participation equity in organised sport and physical activity among children and adolescents in high income countries: a systematic review and meta-analysis
Source: Int J Behav Nutr Phys Act. 2022 Mar 18;19:27. doi: 10.1186/s12966-022-01263-7 (PMC8932332; doi:10.1186/s12966-022-01263-7)
Supplement: Supplementary file 4 — Additional file 4. [file 12966_2022_1263_MOESM4_ESM.docx]

Table 2. Risk of bias within studies

| Study | 1. Were the criteria for inclusion in the sample clearly defined? | 2. Was the sample frame appropriate to address the target population? | 3. Were study participants sampled in an appropriate way? | 4. Was the sample size adequate (i.e., at least 50 participants in each group) ? | 5. Was the response rate adequate, and if 0t, was the low response rate managed appropriately? | 6. Were the study subjects and the setting described in detail? | 7. Was the data analysis conducted with sufficient coverage of the identified sample? | 8. Was the exposure (i.e. SES) measured in a valid and reliable way? | 9. Were the outcomes (i.e. physical activity/ sport) measured in a valid and reliable way? | 10. Was follow up complete, and if not, were the reasons to loss to follow up described and explored? | 11. Were strategies to address incomplete follow up utilized? | 12. Were strategies to deal with confounding factors stated? | 13. Was there appropriate statistical analysis? | Total yes | Total possible | Proportion |
| --- | --- | --- | --- | --- | --- | --- | --- | --- | --- | --- | --- | --- | --- | --- | --- | --- |
| Allen 2015 | 1 | 1 | 1 | 1 | 1 | 1 | Unclear | 1 | 0 | 0 | Unclear | 1 | 1 | 9 | 13 | 69 |
| Amin 2018 | Unclear | 0 | 0 | 1 | Unclear | 1 | 0 | Unclear | 1 | NA | NA | 0 | 0 | 3 | 11 | 27 |
| Andersen 2019 | 1 | 1 | 1 | 1 | 1 | 1 | Unclear | 1 | 0 | NA | NA | 1 | 1 | 9 | 11 | 82 |
| Armstrong 2018 | 1 | 1 | 1 | 1 | 1 | 1 | Unclear | 1 | 0 | NA | NA | 1 | 1 | 9 | 11 | 82 |
| Bagordo 2017 | 1 | Unclear | 1 | 1 | 0 | 1 | Unclear | 1 | 0 | 1 | 0 | 0 | 0 | 6 | 13 | 46 |
| Bann 2019 | 1 | 1 | 1 | 1 | 1 | 1 | 1 | 1 | 0 | NA | NA | 0 | 0 | 8 | 11 | 73 |
| Bengoechea 2010 | 1 | 1 | 1 | 1 | 1 | 1 | 1 | 1 | 0 | NA | NA | 1 | 1 | 10 | 11 | 91 |
| Boone-Heinonen 2010 | 1 | 1 | 1 | 1 | 1 | 0 | Unclear | 1 | 1 | NA | NA | 1 | 1 | 9 | 11 | 82 |
| Borges 2015 | 0 | 1 | 1 | 1 | 1 | 1 | 1 | 1 | 1 | NA | NA | 0 | 1 | 9 | 11 | 82 |
| Bringolf-Isler 2015 | 0 | Unclear | Unclear | 1 | Unclear | 1 | 1 | 1 | 1 | Unclear | Unclear | 1 | 1 | 7 | 13 | 54 |
| Cairney 2015 | 1 | 1 | 1 | 1 | 1 | 1 | Unclear | 1 | 1 | 0 | 0 | 1 | 1 | 10 | 13 | 77 |
| Cameron 2012 | 1 | 1 | 1 | 1 | 1 | 1 | 1 | 1 | 1 | NA | NA | 1 | 1 | 11 | 11 | 100 |
| Carroll-Scott 2013 | 1 | 0 | 1 | Unclear | 1 | 1 | 1 | 1 | 1 | NA | NA | 1 | 1 | 9 | 11 | 82 |
| Clennin 2019 | 1 | 1 | Unclear | 1 | 1 | 1 | 1 | 1 | 1 | 1 | 0 | 1 | 1 | 11 | 13 | 85 |
| Collings 2014 | 1 | 1 | Unclear | 1 | 1 | 1 | 0 | 0 | 1 | NA | NA | 1 | 1 | 8 | 11 | 73 |
| DeCocker 2011 | 1 | 1 | 1 | 1 | 1 | 1 | Unclear | 1 | 1 | NA | NA | 1 | 1 | 10 | 11 | 91 |
| DeCocker 2012 | 1 | 1 | 1 | 1 | 1 | 1 | 1 | 1 | 1 | NA | NA | 1 | 1 | 11 | 11 | 100 |
| Deng 2018 | 1 | 1 | 1 | 1 | 1 | 1 | Unclear | 1 | 1 | NA | NA | 1 | 1 | 10 | 11 | 91 |
| D'Haese 2014 | 1 | 1 | Unclear | 1 | 0 | 1 | Unclear | 1 | 1 | NA | NA | 1 | 1 | 8 | 11 | 73 |
| Dmitruk 2014 | 0 | Unclear | Unclear | 1 | 0 | 0 | 1 | 1 | 0 | NA | NA | 1 | 1 | 5 | 11 | 45 |
| Dollman 2010 | 1 | 1 | 1 | 1 | 1 | 0 | 1 | 1 | 1 | NA | NA | Unclear | Unclear | 8 | 11 | 73 |
| Duncan 2016 | 0 | Unclear | 1 | 1 | 1 | 1 | 1 | 1 | 1 | NA | NA | 1 | 1 | 9 | 11 | 82 |
| Elgar 2016 | 1 | 1 | 1 | Unclear | 1 | 1 | Unclear | 1 | 1 | NA | NA | 1 | 1 | 9 | 11 | 82 |
| Elinder 2014 | 1 | 1 | Unclear | 1 | 1 | 1 | Unclear | 1 | 0 | NA | NA | 1 | 1 | 8 | 11 | 73 |
| Evans 2012 | 1 | 1 | 1 | 1 | 1 | 1 | Unclear | 1 | Unclear | NA | NA | 1 | 1 | 9 | 11 | 82 |
| Fakhouri 2013 | 1 | 1 | 1 | 1 | 1 | 1 | Unclear | 1 | 0 | NA | NA | 1 | 1 | 9 | 11 | 82 |
| Falconer 2014 | 1 | 1 | 1 | 1 | 0 | 1 | 1 | 1 | 0 | NA | NA | 1 | 1 | 9 | 11 | 82 |
| Ferrar 2012 | 1 | 1 | 1 | 1 | 0 | 1 | 1 | 1 | 1 | NA | NA | 1 | 1 | 10 | 11 | 91 |
| Galan 2014 | 1 | 1 | 1 | 1 | 1 | 1 | Unclear | 1 | Unclear | NA | NA | 1 | 1 | 9 | 11 | 82 |
| Goisis 2016 | 1 | 1 | 1 | 1 | 1 | 1 | 1 | 1 | 1 | 1 | 0 | 0 | 0 | 10 | 13 | 77 |
| Gracia-Marco 2010 | 1 | 1 | 1 | 1 | Unclear | 1 | Unclear | 1 | Unclear | NA | NA | 1 | 1 | 8 | 11 | 73 |
| Hardy 2012 | 1 | 1 | 1 | 1 | 1 | 1 | 1 | 1 | 1 | NA | NA | 0 | 0 | 9 | 11 | 82 |
| Heradstveit 2020 | 1 | 1 | 1 | 1 | 1 | 1 | Unclear | 1 | 0 | NA | NA | 1 | 1 | 9 | 11 | 82 |
| Herzig 2012 | 1 | 1 | 1 | 1 | Unclear | 1 | Unclear | 1 | 1 | NA | NA | 1 | 1 | 9 | 11 | 82 |
| Hunt 2019 | 1 | 1 | 1 | 1 | 1 | 1 | 1 | 1 | 1 | NA | NA | 1 | 1 | 11 | 11 | 100 |
| Iguacel 2018 | 1 | 1 | 1 | 1 | Unclear | 1 | 1 | 1 | 1 | NA | NA | 0 | 0 | 8 | 11 | 73 |
| Imhof 2016 | 0 | Unclear | Unclear | 1 | 0 | 1 | Unclear | 1 | Unclear | NA | NA | 1 | 1 | 5 | 11 | 45 |
| Jekauc 2012 | 1 | 1 | 1 | 1 | 0 | 1 | 1 | 1 | 1 | NA | NA | 1 | 1 | 10 | 11 | 91 |
| Jekauc 2013 | 1 | 1 | 1 | 1 | 1 | 1 | Unclear | 1 | 1 | NA | NA | 1 | 1 | 10 | 11 | 91 |
| Jerina 2018 | 1 | 1 | 1 | Unclear | Unclear | 1 | 1 | 1 | 1 | NA | NA | 1 | 1 | 9 | 11 | 82 |
| JimÃ©nez-PavÃ³n 2012 | 1 | 1 | 1 | 1 | 1 | 1 | 1 | 1 | 1 | NA | NA | 1 | 1 | 11 | 11 | 100 |
| Kim 2017 | 1 | 1 | 1 | 1 | 0 | 1 | 1 | 1 | 0 | NA | NA | 1 | 0 | 8 | 11 | 73 |
| Kimbro 2016 | 1 | 1 | 1 | 1 | 1 | 1 | Unclear | 1 | 1 | 1 | 1 | 0 | 1 | 11 | 13 | 85 |
| Kipping 2015 | 1 | 1 | 1 | 1 | 1 | 1 | 0 | 1 | 0 | NA | NA | 1 | 1 | 9 | 11 | 82 |
| Kivimaki 2018 | 1 | 1 | 1 | 1 | 1 | 1 | 1 | 1 | 0 | NA | NA | 1 | 1 | 10 | 11 | 91 |
| Kneeshaw-Price 2013 | 1 | 1 | 1 | 1 | 0 | 1 | 0 | 1 | 1 | NA | NA | 1 | 1 | 9 | 11 | 82 |
| Kobel 2015 | Unclear | 1 | 1 | Unclear | 1 | 1 | Unclear | 1 | 1 | NA | NA | Unclear | 1 | 7 | 11 | 64 |
| Kowaleski-Jones 2017 | 1 | 1 | 1 | Unclear | 1 | 1 | Unclear | 1 | 1 | NA | NA | 1 | 1 | 9 | 11 | 82 |
| Krist 2017 | 1 | 1 | 1 |  |  | 1 |  | 1 | 1 | NA | NA | 1 | 1 | 8 | 11 | 73 |
| Labree 2014 | 1 | Unclear | 1 | 1 | Unclear | 1 | Unclear | 1 | 1 | NA | NA | 1 | 1 | 8 | 11 | 73 |
| Lammle 2012 | 1 | 1 | 1 | Unclear | Unclear | 1 | Unclear | 1 | 1 | NA | NA | 1 | 1 | 8 | 11 | 73 |
| Lampinen 2017 | 1 | 1 | 1 | 0 | 1 | 1 | Unclear | 1 | 1 | NA | NA | 1 | 1 | 9 | 11 | 82 |
| Langlois 2017 | 1 | 1 | 1 | 1 | 1 | 1 | Unclear | 0 |  | NA | NA | 1 | 1 | 8 | 11 | 73 |
| Lehto 2018 | 1 | 0 | 1 | 1 | 0 | 1 | Unclear | 1 | 1 | NA | NA | 1 | 1 | 8 | 11 | 73 |
| Love 2019 | 1 | 1 | 1 | 1 |  | 1 | Unclear | 1 | 1 | NA | NA | 1 | 1 | 9 | 11 | 82 |
| Macniven 2020 | 1 | 1 | 1 | 1 | 1 | 1 | 1 | 1 | 1 | NA | NA | 1 | 1 | 11 | 11 | 100 |
| Manz 2016 | 1 | 1 | 1 | 1 | 1 | 1 | Unclear | 1 | 0 | NA | NA | 1 | 1 | 9 | 11 | 82 |
| McCormack 2011 | Unclear | 1 | 1 | 1 | 1 | 1 | 1 | 1 | 1 | NA | NA | 1 | 1 | 10 | 11 | 91 |
| McNeill 2017 | 1 | 1 | 1 | 1 | 1 | Unclear | 1 | 1 | Unclear | NA | NA | 0 | 1 | 8 | 11 | 73 |
| MiklÃNkovÃ 2016 | 0 | Unclear | Unclear | Unclear | Unclear | 0 | Unclear | 1 | Unclear | NA | NA | 0 | 1 | 2 | 11 | 18 |
| Min 2018 | 1 | 1 | 1 | Unclear | Unclear | 0 | Unclear | 1 | 0 | 1 | Unclear | 0 | 0 | 5 | 13 | 38 |
| Molina-Garcia 2017 | 1 | 1 | Unclear | Unclear | Unclear | 0 | Unclear | 1 | 0 | NA | NA | 1 | 1 | 5 | 11 | 45 |
| Moore 2015 | 1 | 1 | 1 | 1 | 1 | 1 | Unclear | 1 | 1 | NA | NA | 1 | 1 | 10 | 11 | 91 |
| Morgan 2016 | 0 | 1 | 1 | 1 | Unclear | 1 | Unclear | 1 | 1 | NA | NA | 1 | 1 | 8 | 11 | 73 |
| Morley 2012 | 1 | 1 | 1 | 1 | 0 | 0 | Unclear | 1 | 1 | NA | NA | 1 | 1 | 8 | 11 | 73 |
| Mulhall 2011 | 0 | Unclear | Unclear | 1 | Unclear | 1 | 1 | 1 | 0 | NA | NA | 0 | 1 | 5 | 11 | 45 |
| Nielsen 2012 | 0 | Unclear | Unclear | 1 | Unclear | 1 | 1 | 1 | 0 | NA | NA | 1 | 1 | 6 | 11 | 55 |
| Nogueira 2013 | 0 | 0 | Unclear | Unclear | Unclear | 0 | Unclear | 1 | 0 | NA | NA | 1 | 1 | 3 | 11 | 27 |
| Nogueira 2014 | 0 | 0 | Unclear | Unclear | Unclear | 0 | Unclear | 1 | 0 | NA | NA | 1 | 1 | 3 | 11 | 27 |
| Nyberg 2020 | 1 | 1 | 0 | 1 | 0 | 1 | 0 | 1 | 1 | NA | NA | 1 | 1 | 8 | 11 | 73 |
| O'Brien 2020 | 1 | 1 | 1 | 1 | 1 | 1 | 1 | 1 | 0 | NA | NA | 0 | 0 | 8 | 11 | 73 |
| Pabayo 2011 | 1 | 0 | 1 | 1 | 1 | 1 | 1 | 1 | 1 | NA | NA | 1 | 1 | 10 | 11 | 91 |
| Pearce 2019 | 1 | 1 | 1 | 1 | 1 | 1 | 1 | 1 | 1 | 1 | 1 | 0 | 1 | 12 | 13 | 92 |
| Pitel 2013 | 1 | 1 | 1 | 1 | 1 | 1 | 0 | 1 | 1 | NA | NA | 1 | 1 | 10 | 11 | 91 |
| Post 2018 | 1 | 0 | 0 | 1 | Unclear | 1 | Unclear | 1 | 0 | NA | NA | 0 | 1 | 5 | 11 | 45 |
| Poulain 2019 | 1 | 1 | 1 | Unclear | 1 | 1 | Unclear | 1 | 0 | 1 | 1 | 1 | 1 | 10 | 13 | 77 |
| Pouliou 2015 | 1 | 1 | 1 | 1 | 1 | 1 | 1 | 1 | 1 | NA | NA | 1 | 1 | 11 | 11 | 100 |
| PujadasBotey 2016 | 1 | 1 | 1 | 1 | Unclear | 1 | 1 | 1 | 0 | NA | NA | 0 | 1 | 8 | 11 | 73 |
| Puolakka 2018 | 1 | 0 | 1 | Unclear | 1 | 1 | Unclear | 1 | 1 | 0 | 0 | 1 | 1 | 8 | 13 | 62 |
| QunitoRomani 2020 | 0 | 1 | 1 | 1 | 1 | 1 | Unclear | 1 | 0 | NA | NA | 1 | 1 | 8 | 11 | 73 |
| Quon 2015 | 1 | 1 | 1 | 1 | 1 | 1 | Unclear | 1 | 1 | NA | NA | 1 | 1 | 10 | 11 | 91 |
| Rao 2017 | 1 | 1 | 1 | Unclear | Unclear | 0 | Unclear | Unclear | 0 | NA | NA | 0 | 0 | 3 | 11 | 27 |
| Rauner 2015 | Unclear | 1 | 1 | 1 | 1 | 0 | Unclear | 1 | 1 | NA | NA | 0 | 1 | 7 | 11 | 64 |
| Reece 2020 | 1 | 1 | 1 | Unclear | Unclear | 0 | Unclear | 1 | 0 | NA | NA | 0 | 1 | 5 | 11 | 45 |
| Rokicki 2019 | 1 | 1 | 1 | Unclear | 1 | 1 | Unclear | 1 | 0 | NA | NA | 1 | 1 | 8 | 11 | 73 |
| Ruiz 2011 | 1 | 1 | 1 | 1 | Unclear | 1 | Unclear | 1 | 1 | NA | NA | 1 | 1 | 9 | 11 | 82 |
| Salvy 2017 | 0 | 0 | 0 | Unclear | 1 | 1 | Unclear | 1 | Unclear | NA | NA | 1 | 1 | 5 | 11 | 45 |
| Santinello 2012 | 1 | 0 | 1 | 1 | Unclear | 1 | 1 | 1 | 1 | NA | NA | 0 | 1 | 8 | 11 | 73 |
| Shi 2014 | 0 | 0 | 1 | 1 | 0 | 1 | 1 | 1 | 0 | NA | NA | 1 | 1 | 7 | 11 | 64 |
| Sigmundova 2019 | 1 | 1 | 1 | Unclear | Unclear | 0 | Unclear | 1 | 1 | NA | NA | 1 | 1 | 7 | 11 | 64 |
| Smith 2015 | 1 | 1 | 0 | 1 | Unclear | 1 | 0 | 1 | 1 | NA | NA | 1 | 1 | 8 | 11 | 73 |
| Song 2013 | 1 | 1 | 1 | 1 | 1 | 1 | 1 | 1 | 0 | NA | NA | 1 | 1 | 10 | 11 | 91 |
| Steenholt 2018 | 1 | 1 | 1 | 1 | 1 | 1 | 0 | 1 | Unclear | NA | NA | 1 | 0 | 8 | 11 | 73 |
| Tandon 2012 | 1 | 1 | 1 | 1 | 0 | 1 | 0 | 1 | 1 | NA | NA | 1 | 1 | 9 | 11 | 82 |
| Taylor 2020 | 1 | 0 | 1 | 1 | 1 | 1 | Unclear | 1 | 1 | NA | NA | 1 | 1 | 9 | 11 | 82 |
| Uzochukwu 2017 | 1 | 1 | 1 | 1 | Unclear | 1 | 1 | 1 | Unclear | NA | NA | 1 | 1 | 9 | 11 | 82 |
| Vandermeerschen 2015 | 1 | 1 | Unclear | 1 | 1 | 1 | 0 | 1 | 0 | NA | NA | 1 | 1 | 8 | 11 | 73 |
| Vandermeerschen 2016 | Unclear | 1 | Unclear | 1 | 1 | Unclear | Unclear | 1 | 0 | NA | NA | 1 | 1 | 6 | 11 | 55 |
| Vella 2014 | 1 | 1 | 1 | Unclear | 1 | Unclear | Unclear | 1 | 0 | 1 | 1 | 1 | 1 | 9 | 13 | 69 |
| Vermeiren 2018 | 1 | 1 | 1 | 1 | 1 | 1 | Unclear | Unclear | 1 | NA | NA | 1 | 1 | 9 | 11 | 82 |
| Veselska 2011 | 1 | 1 | 1 | 1 | 1 | 1 | 0 | 1 | 1 | NA | NA | 1 | 1 | 10 | 11 | 91 |
| Virtanen 2019 | 1 | 1 | 1 | 1 | 1 | 1 | 0 | 1 | 0 | NA | NA | 0 | 0 | 7 | 11 | 64 |
| White 2012 | 1 | 1 | 1 | 1 | Unclear | 1 | 0 | 1 | 0 | NA | NA | 1 | 1 | 8 | 11 | 73 |
| Wijtzes 2014 | 1 | 1 | 1 | 1 | 1 | 1 | 0 | 1 | 0 | NA | NA | 1 | 1 | 9 | 11 | 82 |
